# SAXS studies of X-ray induced disulfide bond damage: Engineering high-resolution insight from a low-resolution technique

**DOI:** 10.1371/journal.pone.0239702

**Published:** 2020-11-17

**Authors:** Timothy R. Stachowski, Mary E. Snell, Edward H. Snell

**Affiliations:** 1 Hauptman-Woodward Medical Research Institute, Buffalo, New York, United States of America; 2 Department of Cell Stress Biology, Roswell Park Comprehensive Cancer Center, Buffalo, New York, United States of America; 3 Department of Materials Design and Innovation, State University at New York at Buffalo, Buffalo, New York, United States of America; Yale University School of Medicine, UNITED STATES

## Abstract

A significant problem in biological X-ray crystallography is the radiation chemistry caused by the incident X-ray beam. This produces both global and site-specific damage. Site specific damage can misdirect the biological interpretation of the structural models produced. Cryo-cooling crystals has been successful in mitigating damage but not eliminating it altogether; however, cryo-cooling can be difficult in some cases and has also been shown to limit functionally relevant protein conformations. The doses used for X-ray crystallography are typically in the kilo-gray to mega-gray range. While disulfide bonds are among the most significantly affected species in proteins in the crystalline state at both cryogenic and higher temperatures, there is limited information on their response to low X-ray doses in solution, the details of which might inform biomedical applications of X-rays. In this work we engineered a protein that dimerizes through a susceptible disulfide bond to relate the radiation damage processes seen in cryo-cooled crystals to those closer to physiologic conditions. This approach enables a low-resolution technique, small angle X-ray scattering (SAXS), to detect and monitor a residue specific process. A dose dependent fragmentation of the engineered protein was seen that can be explained by a dimer to monomer transition through disulfide bond cleavage. This supports the crystallographically derived mechanism and demonstrates that results obtained crystallographically can be usefully extrapolated to physiologic conditions. Fragmentation was influenced by pH and the conformation of the dimer, providing information on mechanism and pointing to future routes for investigation and potential mitigation. The novel engineered protein approach to generate a large-scale change through a site-specific interaction represents a promising tool for advancing radiation damage studies under solution conditions.

## Introduction

Radiation chemistry in macromolecular X-ray crystallography is a significant issue. When X-rays interact with water, the major component of a crystal or the cellular environment, free radicals are produced. These products consist of solvated electrons (e^-^), hydroxyl radicals (HO^•^), and hydronium ions (H_3_O^+^). The ions can further react with the solvated electrons to produce hydrogen radicals (H^•^) and water [1]. All the radicals cause damage seen in global indicators and as specific structural disruption of individual residues in the resultant model. In the latter case, glutamates and aspartates are decarboxylated, and disulfide bonds and the terminal methyl group on methionines are cleaved. Disulfide bonds and S-methyl thioether groups are the most sensitive to X-ray damage due to the high photo-absorption cross section and electron-affinity of sulfur [2]. This specific damage has garnered much attention due to its potential to misdirect the biological interpretation of protein mechanisms.

Many structural studies have focused on understanding damage mechanisms [3] and mitigating damage through cryo-cooling [4] but also with the use of radical scavenging [5]. Cryo-cooling crystals to ~100 K prevents the diffusion of most solvent generated radicals and only the electrons remain mobile [6]. Damage processes are slowed because the larger radicals are trapped, which can extend the effective crystal lifetime by one to two orders of magnitude [7]. However, cryo-cooling can also limit biologically meaningful conformations [8, 9] and produce structural artefacts [10, 11]. For these reasons there has been interest in a return to near physiological temperature data collection and the corresponding development of serial crystallography methods to limit radiation damage [12].

The X-ray doses used for structural studies are on the order of tens of kilo-grays up to mega-grays and different damage processes have been observed in cryogenic and near physiological temperature crystallographic experiments [7, 12–14]. Damage mechanisms at cryogenic temperature have been well studied at large X-ray doses, but how relevant they are in physiologic conditions (i.e. lower doses, higher temperatures, and in solution) is less well understood.

SAXS is a low-resolution solution technique that can be used to validate the arrangement of proteins observed in crystal structures and develop low resolution models of large protein complexes. It can be used in a more physiologic setting to study large scale conformational changes and dynamics [15, 16]. The radiation dose required to get meaningful data in SAXS experiments is orders of magnitude lower than those used in crystallography. This allows SAXS to be used to study physiologically relevant radiation-protein interactions that are challenging to understand with current biochemical approaches [17].

By engineering a protein that dimerized through a highly solvent exposed disulfide bond, a system is created where disulfide bond breakage leading to monomerization is easily measured by SAXS. In this way, SAXS was able to follow a residue specific rather than a global damage process and link damage pathways seen crystallographically to those occurring in solution with much lower X-ray doses. The X-ray radiation induced dimer to monomer transition was pH dependent, suggesting mechanistic processes, routes for further interrogation, and mitigation strategies.

## Materials and methods

### Protein engineering, expression, and purification

Endoglycosidase-H (endoH) is a 27 kDa globular monomeric protein that is commonly used to deglycosylate proteins to promote crystallization [18] and monitor protein trafficking [19]. It does not contain any native cysteines, is moderately sized, natively monomeric [20, 21] stable under a range of pH values and physiologic temperature [22], and crystallizable [20, 21]. It provides a suitable system to introduce a disulfide to study radiation induced cleavage.

An expression construct was created by fusing endoH (P04067, aa 47–313) to the C-terminus of the maltose-binding protein (MBP) in the pMAL-p5X vector (New England Biolabs; Ipswich, MA) and inserting a tobacco etch virus protease (TEV) cleavage site between the two domains. Previous reports suggested that introducing a disulfide bond is more successful when placed in a flexible region [23] and creates a large loop [24]. Considering this and that the bond should be highly solvent exposed to promote damage and not alter the native folding, we introduced a 7 aa fragment with a single free cysteine (SLSTGCY, 'FRAG_CYS_') attached to the flexible N-terminus of endoH. Cloning was performed by GenScript (Piscataway, NJ). For purification, a His_6_-tag was added to the C-terminus of endoH so that the final construct was MBP-TEV-FRAG_CYS_-endoH-His_6_. For expression, BL21(DE3) cells were transformed with the construct and grown in LB overnight at 30˚C. Cells were diluted to an OD600 of 0.05 and once the OD600 reached 0.3–0.4 the temperature was reduced to 22˚C. At OD600 0.7–0.8 expression was induced with 0.05 mM IPTG and cells continued growing at 22˚C for 24 hours shaking at 250 rpm. Harvested cells were pelleted and lysed in 200 mM NaCl, 20 mM Tris-HCl, pH 7.5, 5 mM imidazole with a microfluidizer. Lysed cells were centrifuged at 60,000 x *g* for 45 minutes and the supernatant was combined with Ni-NTA resin (Marvelgent; Canton, MA) and incubated overnight at 4˚C. The resin was washed with 10 column volumes of 200 mM NaCl, 20 mM Tris-HCl, pH 7.5, 20 mM imidazole and the protein eluted in 8 column volumes of 200 mM NaCl, 20 mM Tris-HCl, pH 7.5, 250 mM imidazole. The eluted protein was incubated with TEV protease (10% w/w) for 60 h at 4˚C. The cleaved protein was separated with a second Ni-affinity purification. Size-exclusion chromatography (SEC) was used as a final purification step and to exchange the protein into 50 mM NaCl, 20 mM Tris-HCl, pH 7.5, 5 mM EDTA. The final dimeric protein is referred to as endoH_CYS_. Disulfide bond formation occurred spontaneously and was monitored by SDS-PAGE and size exclusion chromatography (SEC). Briefly, SEC was performed by equilibrating a Superdex 200 10/300 SEC column (GE Healthcare; Chicago, IL) in 20 mM Tris-HCl, pH 7.5, 50 mM NaCl, 5 mM EDTA. 100 μL of protein at approximately 5 mg/ml was injected for data collection. Data were analyzed using the software Unicorn (GE Healthcare) to obtain the abundance of dimer and monomeric forms. The elution times were compared to a standard curve to determine the corresponding molecular weights. The final protein yield was approximately 65 mg/L.

### Crystallization, data collection, and refinement

Conditions for the crystallization of endoH_CYS_ (10 mg/ml in 20 mM Tris-HCl, pH 7.5, 50 mM NaCl, 5 mM EDTA) were initially determined using a high-throughput microbatch-under-oil method at the Hauptman Woodward Institute High Throughput Crystallization Screening Center [25]. The resulting conditions were optimized, and crystals grown in microbatch under oil by mixing 1 μL protein with 1 μL mother liquor (100 mM TAPS, pH 9, 200 mM magnesium nitrate, and 20% PEG-20,000 (% w/v)) at room temperature. Before cryo-cooling in liquid nitrogen, four rounds of increasing glycerol cryoprotection (30 seconds for each increase of 10%) were carried out in mother liquor.

Cryoprotected crystals were shipped to the Advanced Photon Source at Argonne National Laboratory and diffraction data were collected on a single crystal on beam line 17-ID (IMCA-CAT). The photon energy used was 12.4 keV (1 Å) with data collected on a PILATUS 6M detector at 350 mm distance using oscillations of 0.25° over a 90° rotation range. The data were integrated with MOSFLM [26, 27] and scaling was performed with AIMLESS [28].

Phases were determined using molecular replacement with the structure of monomeric endoH [20] BALBES [29]. The structural model was built using AUTOBUILD [30] and manually extended in Coot [31]. Refinement was performed using an iterative process with PHENIX [32] and Coot. Validation was carried out with MolProbity [33]. The coordinates were deposited as PDB ID 6VE1. Detailed statistics for data collection, processing, and refinement are shown in S1 Table. Structural figures were prepared using PyMOL (Schrödinger; New York, NY) and UCSF Chimera [34].

### SAXS data collection

**Fig 1:**
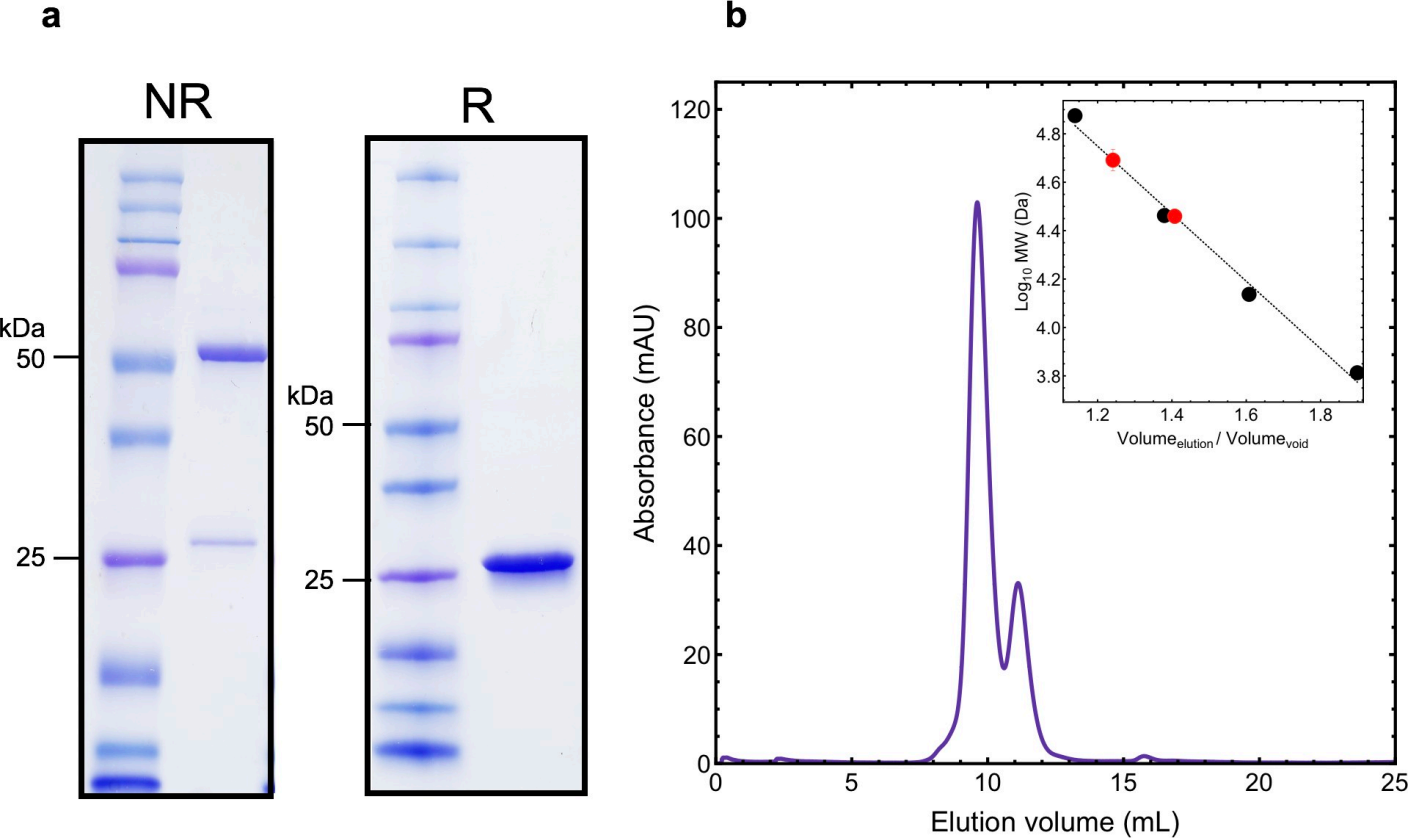
Introducing a cysteine containing fragment leads to disulfide linked dimerization of endoH. In (a) SDS-PAGE analysis shows that the apparent MW of endoH_CYS_ is reduced in the presence of a reducing agent. In (b) SEC analysis shows that about 75% of endoH_CYS_ forms a dimer in solution (b, inset). Comparison of the elution times for each peak with a standard curve shows that the dominant peak has a MW corresponding to the dimer (53 kDa) and the minority peak has a MW corresponding the monomer (28 kDa).

SAXS data were collected using beamline 12.3.1 (SIBYLS) at the Advanced Light Source [35]. The photon energy used throughout was 11.0 keV (1.127 Å). Momentum-transfer values were calculated as *q* = 4πsinθ/λ, where 2θ is the scattering angle and λ is the X-ray wavelength in Å. Data were recorded using a PILATUS 2M detector (Dectris; Philadelphia, PA). A volume of 25 μl of each sample was loaded into the sample chamber. The exposure time for each frame was 0.3 s and a total of 33 frames were collected for each sample in a static position. Buffer from desalting column flow-through was used for matched controls and buffer subtraction. The beamline staff experimentally determined the beam profile (top-hat) and that the flux at the sample was 2.04x10^12^ ph/s was derived based on the scattering of water [36]. RADDOSE-3D modified for SAXS experiments [37] was used for calculating the dose rate (121 Gy/s), taking into account the attenuation (10%) by the sample container (20 μm mica), the beam type (top-hat), and the beam dimensions (3.4 mm^2^). The cell path length was 1.3 mm +/- 0.1 mm. The parameters used for calculating the X-ray dose rate are available in S2 Table and the data collection parameters are summarized in S3 Table. Standard tests were performed to determine how accurately volume fractions could be predicted using the calculated scattering of the dimer and monomer crystal structures. Molecular weight (MW) estimates with SAXS carry an error of about 10% [38]. However, here the predicted molecular weight of the dimer (48.7 kDa) is ~20% lower than the actual MW (60 kDa), while the calculated scattering of the monomer (26.0 kDa) reasonably matches the actual MW (30 kDa). The discrepancy of the dimer MW is explained by the flexibility of the dimer from the engineered linker. MW determination of flexible proteins is a common challenge with SAXS [39] and makes determining experimental volume fractions (VF) difficult. The dimer was designed to be susceptible to X-ray radiation by forming a monomer due to radiation induced cleavage of the covalently bonded disulfide forming the dimer. An experimentally determined dimer and monomer mixture (Fig 1) was used as a starting point to monitor the change in ratio of the two species already known to be present as a function of dose. This reduced uncertainty in comparison between non-irradiated (SEC) and initial irradiated (SAXS) VFs.

The rate of formation of the disulfide radical (a precursor to cleavage) [2] and the type of radicals generated in water-cysteine solutions [40] are pH dependent. To explore the influence of pH on radiation damage, endoH_CYS_ (pI 5.65) was irradiated across a range of pH (5.0, 6.0, 7.5, and 9.0) values while monitoring for structural changes with SAXS. endoH_CYS_, initially purified at pH 7.5, was exchanged into three additional buffers with varying pH values ((1) 20 mM NaC_2_H_3_O_2_, pH 5, 50 mM NaCl, 5 mM EDTA, (2) 20 mM MES, pH 6, 50 mM NaCl, 5 mM EDTA, (3) 20 mM Tris-HCl, pH 7.5, 50 mM NaCl, 5 mM EDTA, or (4) 20 mM Bis-tris propane, pH 9, 50 mM NaCl, 5 mM EDTA) using Zeba desalting columns (Thermo Fisher; Waltham, MA). The samples equilibrated at each pH for approximately one week at 4˚C prior to data collection. Protein concentration was determined from the absorbance at 280 nm and three concentrations (5.0, 2.5, and 1.25 mg/ml) for each pH value were analyzed to determine concentration-dependent effects. Two independent dose series (replicates) at a protein concentration of 5.0 mg/ml for each pH were collected and averaged to improve signal prior to modelling. Scattering of the monomer alone as a control was collected by reducing the disulfide bonds with 2 mM DTT and data was collected similarly to the dimer. All buffer subtracted scattering curves used in the analysis are shown in S1 and S2 Figs.

### Analysis of SAXS profiles

The ATSAS program suite (EMBL) was used for all data analysis [41] except where otherwise noted. *I*(q) error bars and *P*(r) functions were calculated using the GNOM program from ATSAS [41]. Radius of gyration (*R*_g_) values were calculated from the Guinier region with ranges according to *R*_g_*q_max_ ~1.3. To account for the flexible liner, molecular weight (MW) was calculated using the volume of correlation (*V*_c_) method [16], which is more accurate for conformationally dynamic systems than other approaches [15]. Total integrated intensity, singular value decomposition (SVD), elongation ratio (ER), and residuals between experimental and theoretical scattering curves were calculated using custom Python scripts. Volume fraction (*v_k_*) was calculated with a *Mathematica* script according to $\bar{MW}=\sum_{k=1}^{K}v{}_{k}mw_{k}$ and $v_{k}=1=v_{k_{dimer}}+{v_{k}}_{monomer}$ where the MW is 48.7 kDa and 26.0 kDa for dimeric and monomeric forms, respectively, based on the MW estimates for each component from SAXS. Fits of VF trajectories to first order descriptions were performed with a one-phase exponential equation, $v_{f}(d)=v_{f_{0}}e^{-kd}$, where *v_f_*(*d*) is final dose dependent volume fraction, $v_{f_{0}}$is the initial volume fraction, *k* is the rate constant (Gy^-1^), and *d* is the X-ray dose (Gy). This was performed in *Mathematica* using the NonlinearModelFit function. Calculated scattering from crystal structures was calculated with CRYSOL [42]. To follow the fragmentation trajectory, OLIGOMER [43] was used to determine the fit and volume fraction of dimer and monomer components to a series of experimental scattering data. The monomer component was based on the first exposure (36.3 Gy) of the experimental monomer at pH 7.5 and 5.0 mg/ml protein concentration and the dimer component was developed by subtracting the volume fraction weighted monomer component from the first exposure (36.3 Gy) of experimental scattering of the dimer-monomer mixture also at pH 7.5 and 5.0 mg/ml concentration. *Ab initio* electron density reconstruction was performed with DENSS, an algorithm particularly suited to potential dynamics present in the system using the dimer (monomer subtracted) scattering curve [44]. The characteristics calculated for the data series used for modelling are summarized in S4 Table.

### Ensemble optimization

The disulfide forming fragment was designed to be flexible, and likely to sample many conformations in solution. Therefore, the ensemble optimization method (EOM) [45] was employed. This approach can generate models with different conformations between the monomer domains, can find an ensemble of conformations that together best explain the experimental scattering data, and is sensitive to any conformational distribution of the dimer over the course of irradiation.

Data used for modelling was collected close to physiologic conditions at pH 7.5. To improve signal-to-noise, two identical replicate dose series at pH 7.5 and 5.0 mg/ml concentration were averaged and used for modelling. EOM was performed with monomeric endoH [20] as a rigid body. To simulate a disulfide bond between two endoH monomers a protocol was adapted from Tian *et al*. [46] where a Cys-Cys fragment was extracted from PDB entry 1HZH and treated as another rigid body. The program RANCH from the EOM package [47] was then used to generate 10,000 random conformations of a linker between each endoH and the Cys-Cys fragment, thereby simulating a hinge motion. The final pool contained 10,000 monomeric and 10,000 dimeric structures. The optimized ensemble was selected using the genetic algorithm (GA) in the program GAJOE [48] and was repeated 100 times.

## Results

### Engineering and crystal structure of an X-ray cleavable disulfide linked dimer

Soluble endoH_CYS_ was produced. Analysis of the mobility shift on SDS-PAGE in the presence of a reducing agent showed that the protein migrated to a lower MW, indicating the presence of inter-molecular disulfides (Fig 1A and S3 Fig). Comparison of the elution profile of endoH_CYS_ in size-exclusion (SEC) chromatography shows that transient disulfide bond formation leads to dimerization in approximately 75% of endoH_CYS_ (60 kDa theoretical, 53 kDa experimental) in solution (Fig 1B).

**Fig 2:**
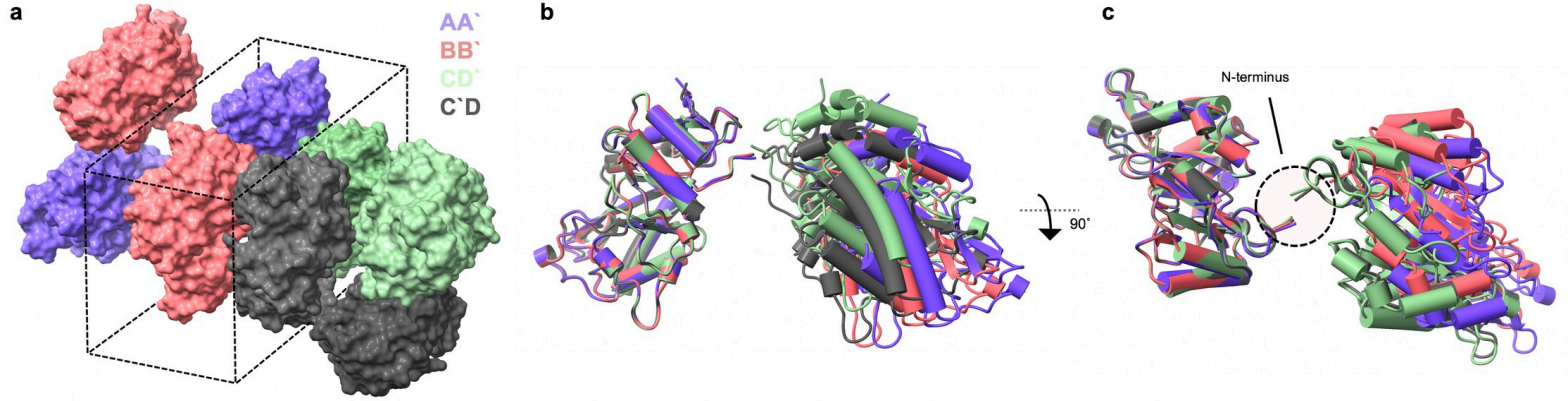
Crystal structure of dimeric endoH_CYS_ suggests a highly exposed and flexible disulfide bond linkage. In (a) the crystal packing diagram showing that the asymmetric unit contains four monomers with the suggested dimers illustrated with chains that have matching color. (b) A front view of an alignment of one chain from each dimer is shown with the (c) top view of the alignment indicating the position of the N-terminus and disulfide linkage. The slight differences in structure of the individual dimers are seen by the non-ideal alignment.

A structural model was determined to a resolution of 2.1 Å by X-ray crystallography to determine the orientation of the dimer (PDB 6VE1) (Fig 2). The protein crystallized in space group P2_1_22_1_ with four monomers in the asymmetric unit (Fig 2A). The monomers have an average RMSD 0.289 +/- 0.016 Å to the original monomeric structure [20], indicating that the cysteine containing fragment did not affect folding. A PISA [49] analysis of the interfaces within the asymmetric unit suggests that there are too few interactions between crystal packing oligomers to for those interactions to form in solution. The cysteine containing fragment and disulfide bond are un-resolved in the electron density but is not unexpected due to the deliberate placement of the disulfides in an accessible and already flexible region. The position of the N-terminus indicates that the disulfide linked dimer forms between monomers across adjacent asymmetric units, yielding dimers with chains AA', BB', CD', and C'D (Fig 2B). This arrangement with the N-terminus of adjacent monomers positioned in close proximity is not seen in crystal structures of monomeric endoH [20, 21]. The distance between the modeled N-terminal Val-8 on partnered monomers (13 Å for BB' and AA', 7 Å for both CD dimers) is such that the length of the introduced fragment can satisfy the gap and connect the monomers (Fig 2C). Aligning the dimers shows slight differences in the inter-monomer orientation, which is most likely due to crystal packing (Fig 2B).

### X-ray radiation drives protein fragmentation in solution experiments

**Fig 3:**
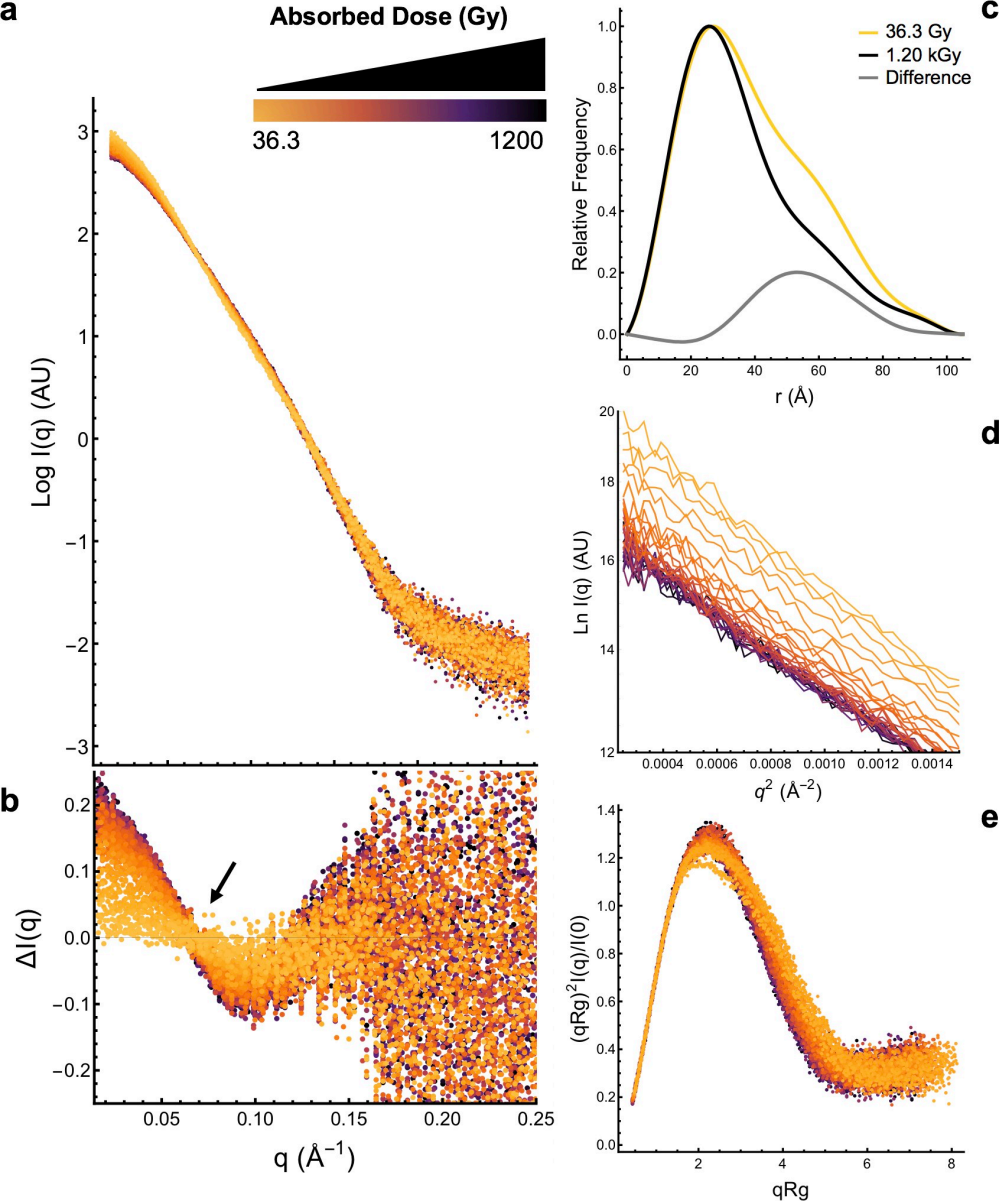
X-ray solution scattering analysis indicating that fragmentation is radiation dose dependent. The color gradient corresponds to the magnitude of the absorbed dose (36.3 Gy-1.2 kGy) delivered across 33 x 0.3 sec exposures where yellow is low dose and black is high dose. EndoH_CYS_ was irradiated at pH 7.5 and the results shown are averages of two replicates at a concentration of 5.0 mg/ml. In (a) buffer subtracted scattering curves exhibit a dose-dependent decrease in intensity. In (b) residuals calculated between the first exposure and each sequential exposure show that intensity decreases at low-*q* and increases at high-*q* with increasing absorbed dose. The arrow identifies an isoscattering point at *q* ~ 0.07 Å^-1^, characteristic of a transition between two states. In (c) a pairwise distance distribution plot, *P*(r), normalized to MW, of the first, last exposure, and difference between first and last exposure shows that the fraction of longer distances in the protein decreased. The slope of the Guinier region remains linear (d) but decreases, indicating that the size of the protein is decreasing. A Dimensionless Kratky plot (e) also indicates that the protein is changing shape during irradiation.

The quality of SAXS data from globular monomodal samples can be evaluated by various quality criteria [50]. In our case we expect a mixture of dimers and cleaved monomer formation so these criteria are not suitable as quality indicators but can be explored to monitor the dimer to monomer progression. The fundamental evidence of radiation damage in SAXS is seen through changes in the scattered intensity. An increase in scattering at low-*q* indicates aggregation and a decrease indicates fragmentation [51]. For the endoH_CYS_ irradiated at four concentrations at pH 7.5, there was no evidence in the data indicating buildup of damaged protein on the sample cell windows over time. There was a dose-dependent decrease in intensity at low-*q* (*q* ~ 0.01–0.07 Å^-1^) and an increase beginning at mid-*q* (*q* > ~ 0.07 Å^-1^) that was noticeable between the first (36.3 Gy) and second exposure (72.6 Gy). This change in the shape of the scattering curve is characteristic of fragmentation (Fig 3A and 3C). An isoscattering point was observed at *q* ~ 0.07 Å^-1^ indicating a transition within a system consisting of only two components [52]. This was predicted based on the theoretical scattering of the monomer and dimer crystal structures (S4 Fig) and confirms fragmentation of the dimer. The difference in the location of isoscattering point in calculated and experimental scattering curves suggests that the conformation of the dimer is different in solution, perhaps through freedom crystal lattice restrains to rotate around the flexible linker. The intensity in the Guinier region exhibited a dose dependent decrease while remaining linear (Fig 3D). The slope of the Guinier region is related to the radius of gyration (*R*_*g*_). Although dose does not affect the linearity of the Guinier region, the initial *R*_*g*_ (36.3 Gy) was 30.6 Å and decreased after the total accumulated dose (1.2 kGy) to 27.2 Å (S4 Table). The *R*_*g*_ calculated from the theoretical scattering of monomeric endoH and the average of dimers from the crystal structure (Fig 2 and S4 Fig) are 19.4 and 31.8 +/- 1.65 Å, respectively. A dimensionless Kratky plot provides a semi-quantitative approach to assessing protein change that is normalized for differences in particle mass [53]. As endoH_CYS_ receives more radiation, the bell-shaped intensity curve in the Kratky plot becomes taller and narrower indicating a change in sample shape (Fig 3C). The elongation ratio (ER), which is calculated from the *P*(r) curve, estimates protein compactness where ER ~ 1 is compact and ER >> 1 is elongated [54]. The ER for endoH_CYS_ becomes smaller at higher doses (ER_36.3 Gy_ = 2.17 and ER_1.2 kGy_ = 1.91) also indicating the protein is shifting to a more compact state. This change in shape is also reflected in the loss of longer distances in the 30–80 Å range (Fig 3C). Controls with the monomer (S2 Fig) and buffer (S5 Fig) did not yield dose-dependent changes. Together, these results reveal a two-component system with a dose dependent transition from the flexible disulfide linked dimer to a compact monomer. Fragmentation occurs without any evidence of aggregation or inter-particle interactions in the Guinier region (Fig 3D).

### Damage pathway is pH dependent

**Fig 4:**
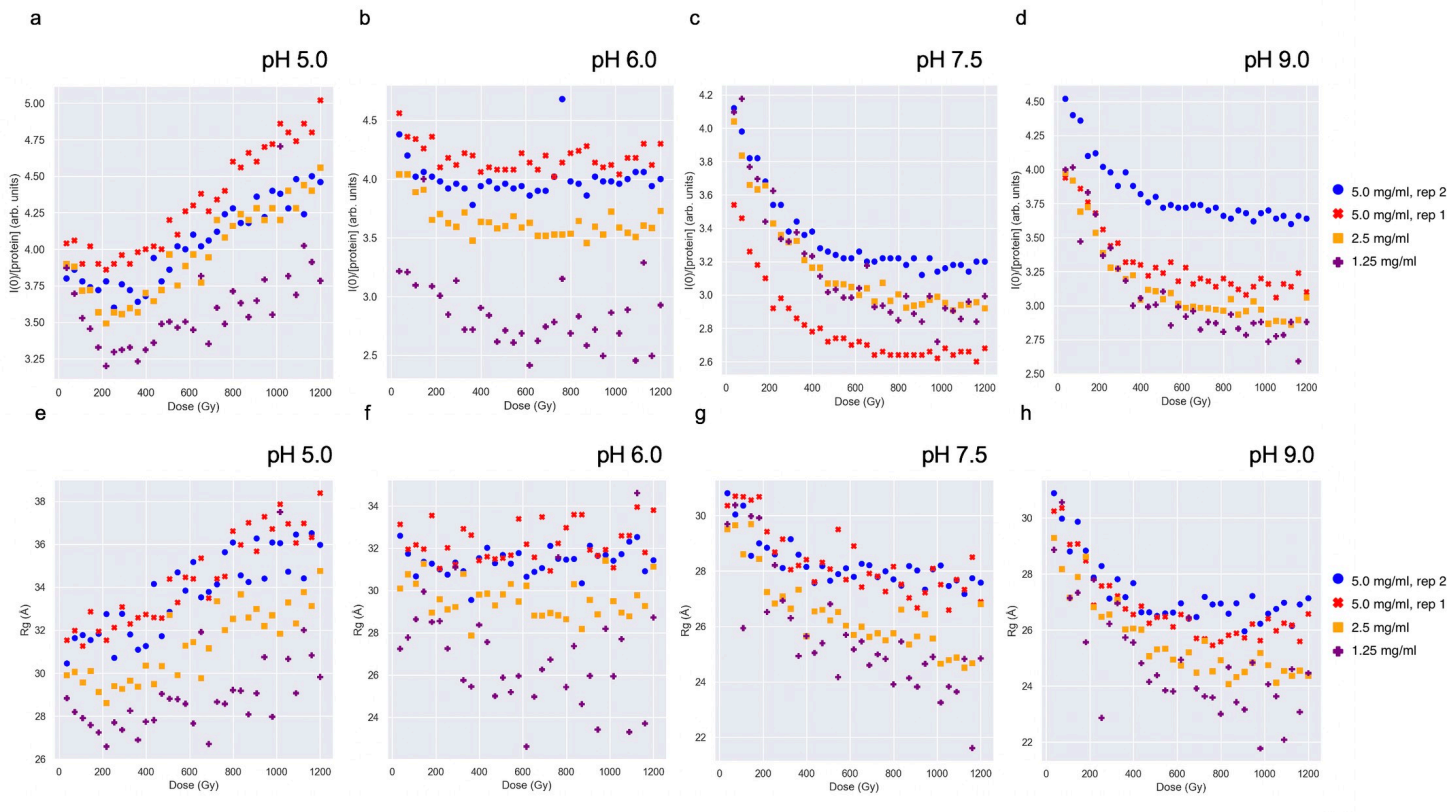
Fragmentation and aggregation damage pathways are pH and concentration dependent. Samples were irradiated at four pH values (5.0, 6.0, 7.5 and 9.0) and three concentrations each (5.0, 2.5, and 1.25 mg/ml). Experiments at 5.0 mg/ml were repeated twice with fresh sample. In (a-d) *I*(0) and (e-h) *R*_g_ values for each experimental condition were calculated with AUTORG [55]. *I*(0) values were normalized by dividing by the concentration.

The fragmentation observed in SAXS (Fig 3) indicates disulfide bond cleavage occurred from radiation exposure. To explore the role of pH on radiation damage, additional SAXS experiments were performed where endoH_CYS_ (pI 5.65) was irradiated with SAXS at three additional pH values (5.0, 6.0, and 9.0) and three concentrations and compared to the previous data that was collected at pH 7.5 (Fig 3). The results indicate that the magnitude and direction of the change in the intensity at zero scattering angle, *I*(0), were strongly pH dependent (Fig 4). Until 254 Gy, samples at each pH exhibited a decrease in *I*(0) agreeing with fragmentation, but the magnitude of the change increased with increasing pH values. Specifically, at the total accumulated dose (1.2 kGy), the overall decrease in *I*(0) at pH 6.0 was much less pronounced than at pH 7.5 and 9.0, while pH 5.0 showed an overall increase in *I*(0) (Fig 4A–4D). The increase at pH 5.0 indicates aggregation and was not apparent at the other pH values. Similar results were seen in *R*_g_ (Fig 4E–4H). At a particular pH, a similar process was seen across concentrations but the magnitude varied. At pH values where fragmentation occurred (i.e. pH 6.0, 7.5, and 9.0) fragmentation appeared greatest at low concentrations. At pH 5, which predominately aggregated, the increase in *I*(0) and *R*_g_ was greater at higher concentrations. Different structural trajectories at high and low pH are also indicated by opposite shifts in respective Kratky plots (S6 Fig). The initial scattering of the dimer component at each pH were overall similar indicating these changes were in part radiation driven (S7 Fig). The scattering of the monomer component alone showed a slight dose dependent increase in *I*(0) and *R*_g_ at low pH but was largely unaffected by radiation (S8 Fig). These results indicate that different damage mechanisms (fragmentation and aggregation) occur in the same sample with the same radiation dose but at different solution pH values. Altering the solution pH will affect the surface charge of the protein and the composition of the solvent. The aggregation at pH 5.0 and fragmentation at pH 6.0 span the isoelectric point, 5.65. The different results suggest a residue specific effect with the consequence that aggregation caused by radiation effects might be mitigated by adjusting the pH of the buffer.

**Fig 5:**
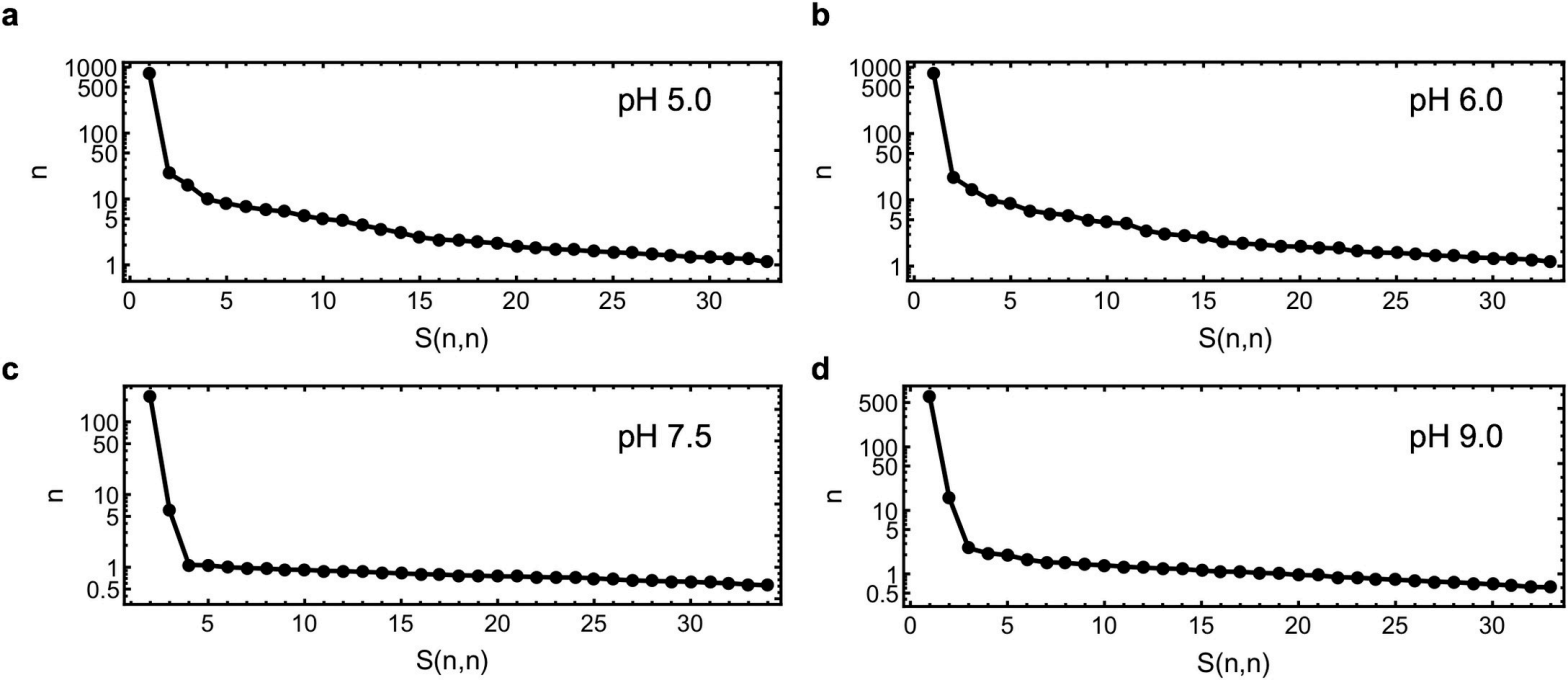
Fragmentation includes two components. The horizontal units are arbitrary. Singular value decomposition analysis (SVD) was conducted on data at 5.0 mg/ml for each pH value. In (a) pH 5.0 and (b) 6.0 shows the scattering is from multiple components, which is characteristic of aggregation. SVD at (c) pH 7.5 and (d) pH 9.0 shows the scattering is from two components, suggesting a process with only monomer and dimer components.

Single value decomposition (SVD) allows the number of distinct meaningful components that contribute to a series of data to be estimated [56]. An SVD analysis of the dose series of scattering data shows that endoH_CYS_ pH 5.0 and 6.0 contain multiple components (with values greater than zero) that contribute to the overall scattering (Fig 5A and 5B). This is characteristic of aggregation where many particles of different sizes are generated through non-specific radical induced cross-linking [57]. Interestingly, while pH 6.0 exhibited an initial intensity decay indicating fragmentation, the fragmentation is greatly reduced compared to pH 7.5 and 9.0 (Fig 4B). The SVD analysis shows multiple scattering components at pH 6.0 while only two at pH 7.5 and 9.0 (Fig 5B and 5C). This suggests that the reduced fragmentation at pH 6.0 is caused by underlying and simultaneously occurring aggregation that results in a less substantial decay in *R*_*g*_ and *I*(0) as a function of X-ray dose (Fig 3). SVD estimated that the scattering of the monomer alone (S2 Fig) at each pH contained an equal number of components (S9 Fig) and validates that the differences observed in the dimer-monomer mixture between high and low pH are due to different damage processes specific to the dimer. Importantly, the two components present at high pH agree with a dimer to monomer transition through a disulfide bond cleavage.

### A dimer to monomer transition explains fragmentation

**Fig 6:**
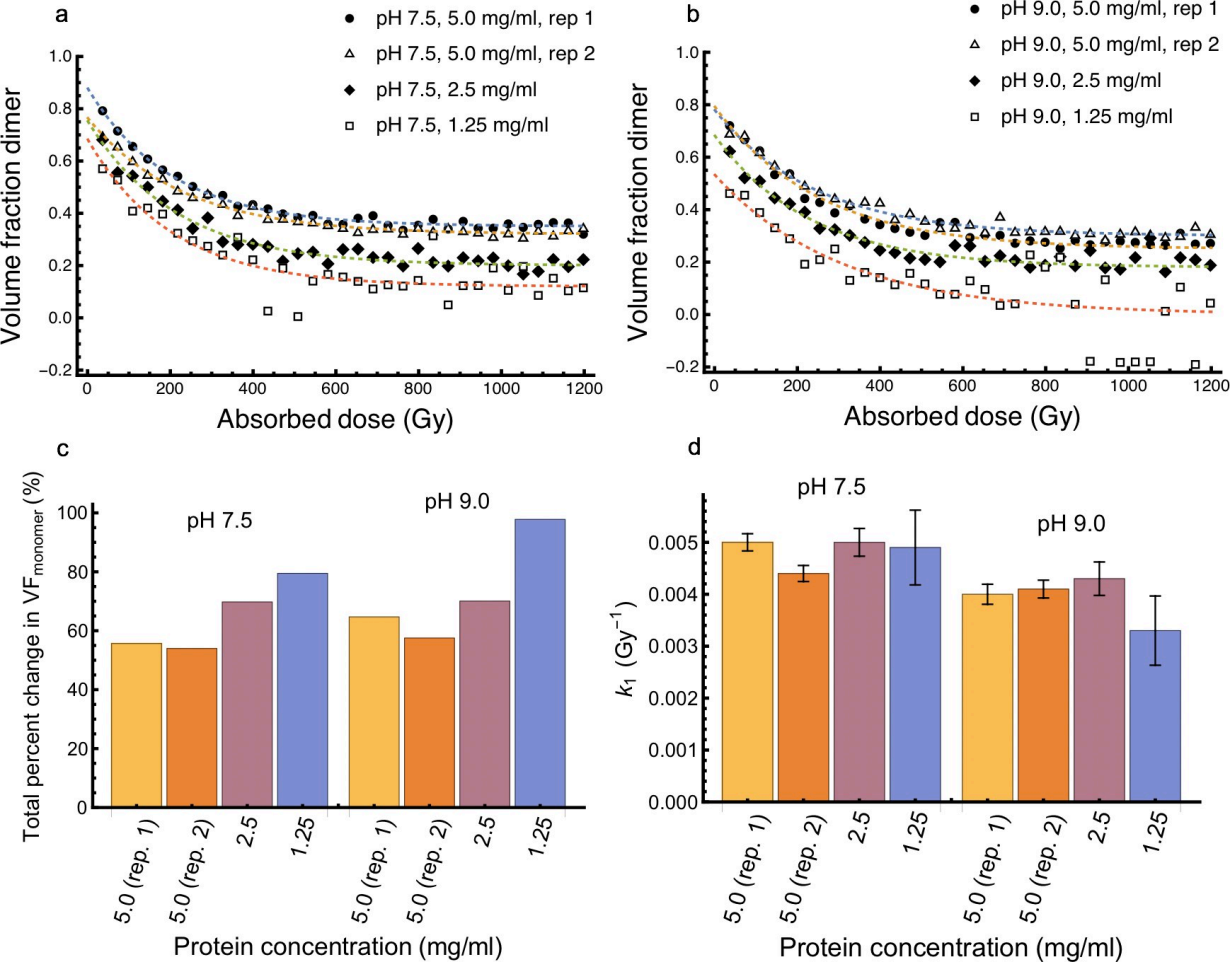
The fragmentation processes can be modelled by a dimer to monomer transition and follows first-order kinetics. The volume fraction (VF) of monomer and dimer components were estimated based on the MW using the volume of correlation, *V*_*c*_, method. The MW of monomer and dimer components were based on the experimental scattering of the monomer and calculated scattering of the dimer. VF analyses were performed at pH 7.5 (a) and 9.0 (b). In (c) The total percent change in monomer was calculated using the VF of monomer at the first exposure (36.3 Gy) and the last exposure (1.2 kGy). In (d) The rate constants, *k*_1_, were calculated by fitting the data to one-phase exponential decays. Error bars represent the standard error in approximating the rate constants.

The samples at pH 7.5 and pH 9.0 at 5.0 mg/ml concentration included two components based on the SVD analysis (Fig 5) and enabled a volume fraction (VF) analysis to be performed. The multiple components present in samples irradiated at pH 5.0 and 6.0 prevented a VF analysis. The average molecular weight of the monomer and dimer components were calculated (volume of correlation, *V*_*c*_) [16] using the experimental monomer scattering (S10 Fig) and calculated scattering of the dimer structure (S4 Fig). The VF analysis shows that at both pH 7.5 and 9.0 the protein is roughly 25% monomer and 75% dimer after the first exposure of 36.3 Gy, which is in agreement with the experimental SEC analysis (Figs 1B and 6B). The VF trajectories suggests that the rate of fragmentation decreases as dose accumulates (Fig 6A and 6B). This indicates that the rate is proportional to the amount of dimer remaining in solution, which reflects a first order process and is a common description for many chemical and biological processes. The fit of the VF trajectories to a first order exponential equation indicates that the total amount of fragmentation was greater as concentration decreased (Fig 6C and S5 Table). The rate of fragmentation was consistent across concentration and slightly reduced at pH 9.0 compared to pH 7.5 (Fig 6D). However, this difference in the rate of fragmentation did not lead to a systematic change in the magnitude of fragmentation between the two pH values (Fig 6C). Interestingly, the sample never transitions to an entirely monomeric system but reaches an equilibrium at approximately 600 Gy in spite of additional X-ray doses.

Collecting experimental data for the dimer alone is challenging as a mixed dimer/monomer system is created from the initial exposure (Fig 4). While a calculated curve from the crystallographic model could be used, the dimers in the asymmetric unit differ through small inter-monomer rotations, and that difference is distinguishable in their calculated scattering curves (S4 Fig). To overcome this, an experimental curve for the dimer was obtained by subtracting the volume fraction weighted contribution (based on MW) of the experimental scattering of the monomer from the experimental scattering of the mixture at the first exposure (36.3 Gy). Comparison of the monomer subtracted scattering (dimer) to the theoretical scattering of the crystal structure dimers yielded reasonable agreement at low-*q*, validating the approach (S11A Fig). The resulting electron density reconstruction supports a dimer but in a slightly different orientation to that seen in the crystallographic model (Fig 2 and S11B and S11C Fig). This electron density model was consistent with one produced from a conventional bead modelling approach (S12 Fig). That the solution structure is distinct from the crystal structure is not unexpected given the removal of crystal packing constraints and the intended flexibility of the designed linker.

**Fig 7:**
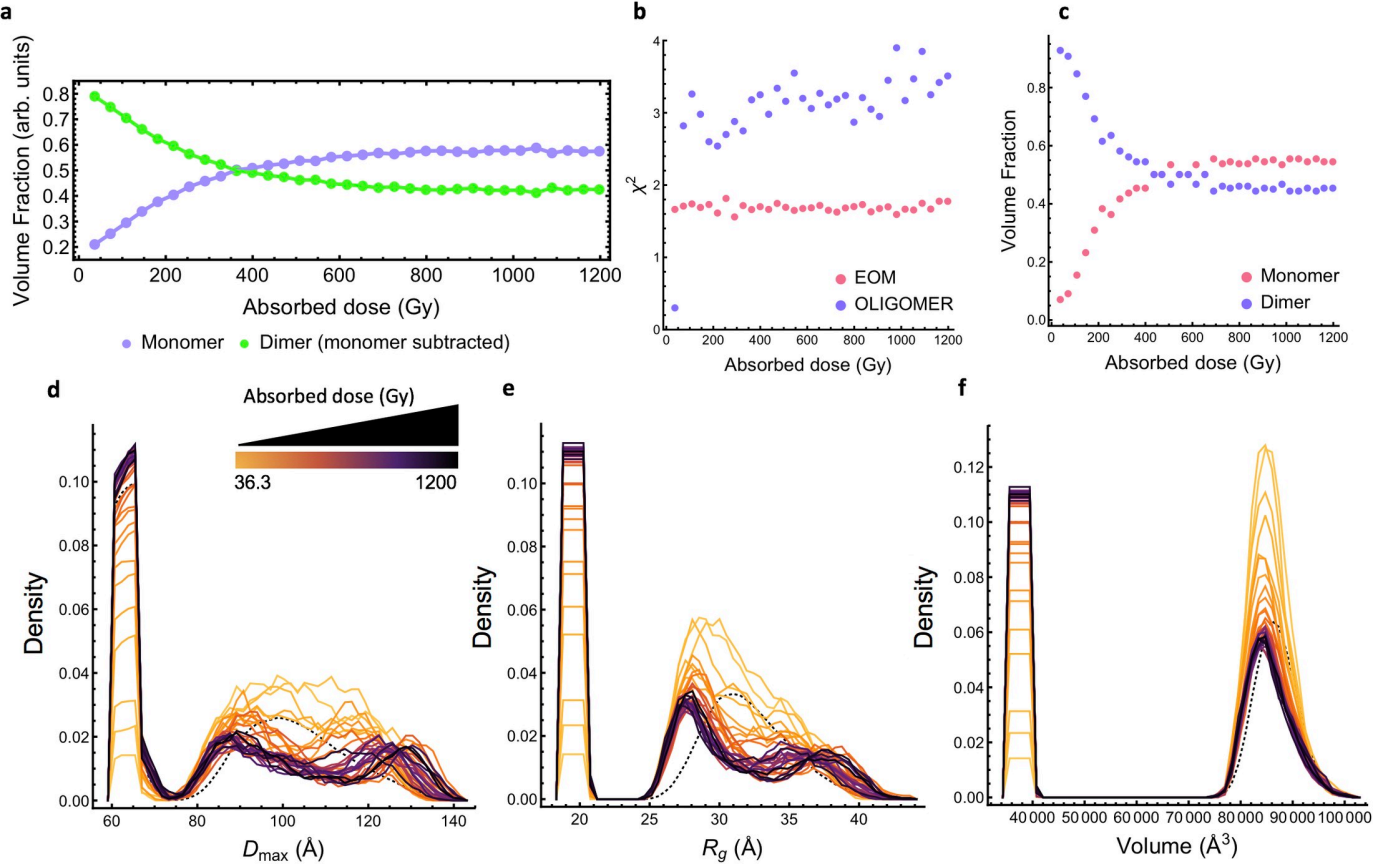
Radiation drives a change in the magnitude and conformation distribution in the engineered dimer. In (a) the program OLIGOMER [43] was used to calculate the volume fraction of monomer and dimer components considering the scattering curve of each component. The first exposure (36.3 Gy) of the experimental scattering of the monomer at pH 7.5 and 5.0 mg/ml served as the monomer component and the dimer component was developed by subtracting the VF weighted contribution of the monomer component from the first exposure (36.3 Gy) of experimental scattering of the mixture. In (b) *χ*^2^ values show that the (pink) EOM approach explains the data better than the OLIGOMER approach (purple). In (c) the volume fractions calculated from the EOM results that follow the fragmentation process. Population results from the EOM analysis are shown in (d) with the distribution of *D*_max_, (e) *R*_g_, and (f) volume of the selected ensembles. Dotted line represents the random pool. The color gradient corresponds to the magnitude of the absorbed dose (36.3 Gy-1.2 kGy) delivered across 33 x 0.3 sec exposures where yellow is low dose and black is high dose.

An additional VF analysis was performed for the data closest to physiological conditions, pH 7.5, using the scattering curve of the monomer subtracted (dimer) and the experimental monomer scattering as additional restraints. This analysis was in good agreement with the previous VF analysis based on MW alone (Figs 6A and 7A). While the fits to the experimental data were reasonable, they worsened at higher doses, as evidenced by the χ^2^ values (Fig 7B). The monomer subtracted scattering represents the dimer well at low doses, but it becomes less accurate at higher doses (Fig 6A). The experimental scattering of the monomer component alone did not change during irradiation (S8 Fig) and suggests that the conformation of the dimer changes during irradiation.

To account for possible changes in conformations, the fragmentation series at pH 7.5 were analyzed using the ensemble optimization method (EOM) [45]. The resulting EOM models ($\bar{\chi^{2}}∼1.65$) to the experimental data was greatly improved compared to fits with the crystal structures ($\bar{\chi^{2}}∼4.00$), especially at high-*q* (Fig 7B and 7C and S13 Fig). The volume fraction analysis from EOM is also in reasonable agreement with the previous approaches, showing that the monomer fraction becomes the dominant species at higher doses (Figs 6, 7A and 7C). Most ensembles chosen by the genetic algorithm contained on average 15 models, with the largest containing 20, validating the assumption about conformationally flexibility. This is also confirmed by analysis of the resulting ensemble distributions. The ensemble of the first exposure (36.3 Gy) is more flexible than the random pool (*R*_flex-ens_/*R*_flex-pool_ = 84.04% / 72.14%). After the total accumulated dose (1.2 kGy) the ensemble is as flexible as the random pool (*R*_flex-ens_ = 72.14%). This agrees with the Kratky plot and *P*(r) distribution that indicated that the protein became more compact with increasing doses of X-rays (Fig 3). This is most likely due to the generation of monomers that are less flexible than the linked dimer and are predominate at higher doses. However, as the sample receives more dose, the distribution of *R*_g_ and *D*_max_ of the dimer population also changes suggesting that radiation drives a change in both the magnitude and conformational distribution of the dimer. Specifically, at low doses the distributions of both *R*_g_ and *D*_max_ are somewhat monomodal and centered relative to the random pool but as dose increases both transition to a bimodal distribution. The sample loses moderate (~33 Å and ~115 Å) conformations and becomes enriched with both compact (~27.5 Å and ~87.5 Å) and extended conformations (~37 Å and ~130 Å) (Fig 7D–7F and S14 Fig). Fitting the dose-dependent changes at these positions to a logarithmic regression shows that for *R*_g_ the middle portion of the peak (33 Å) decreases more quickly (-0.237 density (arbitrary units)/Log(Gy)) than the extended (37 Å; -0.029 density/Log(Gy)) and compact portions (27.5 Å; -0.116 density/Log(Gy)) (S14A Fig and S1 Movie). This was also observed for *D*_max_ where mid-length distances (115 Å) decreased faster at a rate of -0.292 density/Log(Gy)) compared to extended (130 Å; 0.090 density/Log(Gy)) and compact (87.5 Å; -0.091 density/Log(Gy)) distances (S14B Fig and S2 Movie). These results suggest that in addition to changes in the magnitude of the dimer population, the conformation distribution of the dimer also changes over the course of irradiation which could be interpreted as a conformation dependence on damage susceptibility. These dose driven changes in *R*_g_ and *D*_max_ distributions of the dimer were similar at pH 7.5 and 9.0 (S15 Fig).

## Discussion and conclusion

The overall goal of our study was to relate damage seen in the crystallographic state to that observed in solution, specifically X-ray induced damage to the sensitive disulfide bond (cystine). Disulfides are particularly susceptible to radiation damage due to the high electron affinity of sulfur. They are attacked by solvated electrons generated as part of the photolysis of water and these electrons are mobile even at cryogenic temperatures [2]. Under cryogenic conditions and crystallographic doses as low as 5 kGy, the disulfide bond suffers reductive damage [58]. Weik *et al*. [59] have explored disulfide damage below (100 K) and above (155 K) temperatures where the larger free radicals are mobile. Structural changes to the disulfide position were seen at 155 K but not at 100 K, and the disulfides were highly radiation sensitive at 155 K. This extends to the solution case where in addition to the solvated electrons, disulfides can also be impacted by radicals that are otherwise cryogenically trapped. Radiation damage to cysteine has been studied in solution for decades but the reaction mechanism for the diverse array of products are not fully understood [60]. The results presented here cannot specifically determine or quantitate the reactions with the larger radicals trapped in the cryogenic state but do provide qualitative evidence that they are occurring.

In our case changes in the scattering curve that are characteristic of fragmentation are observed with doses less than 100 Gy, fifty times less than the crystallographic studies [59]. Fragmentation is confirmed by changes in basic parameters such as decreases in *R*_g_ and *I*(0) (Fig 4). SVD analysis shows fragmentation includes a two-component system, which supports fragmentation through disulfide bond cleavage rather than main chain breakage (Fig 5). These results from SAXS are compatible with data from the large number of solution studies on cysteine and damage seen in the crystallographic case. Here, no sample reached a completely monomeric state and the rate of monomerization decreased as the absorbed dose accumulated (Fig 6). Irreversible bond breakage is favored where the bond rupture causes a positional shift [61]. Incomplete monomerization could be explained by the presence of repair pathways, which can prevent bond breakage leading to fragmentation but is unlikely to lead to re-association of previously fragmented monomers. A repair pathway occurs in crystallographic studies, but it was not seen to have a noticeable impact [58]. Damage to disulfide bonds is not uniform and can vary with the immediate environment in the protein. The linker connecting the dimer was designed to be flexible and therefore the dimer is most likely sampling conformations in solution (Fig 2). Analysis of ensemble modelling reveals that the conformation of the dimer changes as dose accumulates (Fig 7). This indicates that the local bond environment differs within the conformational ensemble and despite the solvent accessibility, may have some impact on the incomplete fragmentation observed. This is consistent with crystallographic studies where disulfide orientation and solvent exposure influence radiation damage susceptibility [62]. The EOM results are not precluded by the SVD analysis that suggests that there are only two components in solution (Fig 5). While SVD is a powerful method it has weaknesses. SVD describes the minimum number of components necessary to explain the experimental data. As others have noted, it might not identify all states that exist. This is particularly problematic for studies on disordered systems or kinetics experiments with short exposures [63–65], which is the focus of this study.

The rate of formation of the disulfide radical (a precursor to cleavage) [2] and the type of radicals generated in water-cysteine solutions [40] are pH dependent. The susceptibility of disulfides to cleavage is also influenced by local conditions such as pH [2, 4, 66]. Our results with pH values spanning the isoelectric point (pI ~ 5.65) all yielded initial changes in the *I*(0), *R*_g_, and MW that are indicative of fragmentation, which accelerated as pH increased (Fig 4). Fragmentation appeared inversely related to concentration where fragmentation was greater at lower concentrations (Fig 6). However, in contrast to global damage [57], fragmentation was not entirely dose dependent as it reached an equilibrium prior to complete monomerization (~ 600 Gy) in spite of additional X-ray doses (Fig 6). It is unclear if this plateau effect, which could be described by first order kinetics, reflects the concentration dependence of the underlying solution chemistry or the protein.

At pH 6.0 (near the isoelectric point), the change in intensity was small and remained within 10% of the initial values (Fig 4) and could be due to the presence of aggregating species as suggested by the SVD analysis (Fig 5). The magnitude of fragmentation was similar at pH 7.5 and 9.0 but much greater than at pH 6.0 (Fig 6). By necessity each pH condition was prepared with different buffering agents since there are no single biologic buffers that function over the wide range of pH studied. These factors add additional layers of complexity to the analysis but shows that the solution pH whether through hydrogen ion activity, chemical properties, or both, influences both global and a specific radiation damage mechanism, aggregation and fragmentation, in SAXS.

Since thiol groups have a pK_a_ of 8.3 and 8.5 disulfides are be more stable at higher pH values [66]. The protein was initial purified at pH 7.5 so that each sample should at least 75–80% dimer based on the SEC analysis (Fig 1) and as covalent bonds, they are unlikely to be reversed by subsequent changes in the pH of the buffer. However, initial estimates of the dimer VF decreased with concentration (Fig 6). It is possible that a small population of dimers formed through electrostatic interactions, which are known to be influenced by both pH and protein concentration. The presence of non-covalently associated dimers might also explain the increased propensity for aggregation that was observed at low pH as well as the fragmentation resistant population of dimers (Fig 7). However, another explanation is that the discrepancy in initial VF estimates between concentrations is due to differing degrees of damage that occur within the first exposure. Lower protein concentrations exhibited greater total fragmentation than higher protein concentrations at the same pH (Fig 6). Since fragmentation was apparent between the first and second exposures (Fig 3), it is likely that fragmentation also occurs at doses lower than the initial dose. How additional factors such as X-ray dose-rate or diffusion rate influence disulfide bond cleavage in solution are not examined by this study. The pathways for damage are more complex in solution due to the free motion of radical species that are otherwise cryogenically trapped. Serial crystallography studies of lysozyme show that global damage occurs at five times the rate of specific damage for crystals grown at pH 4.5 [12]. Other studies at slightly higher pH and different experimental conditions suggest a lower rate [14]. Comparing the crystallographic results to the solution case for disulfide damage is difficult. Typically, SAXS is only sensitive to global damage through changes in the scattered intensity. Here, our system is engineered so that the reduction in scattered intensity is from the dimer to monomer transition through disulfide bond cleavage, making a global damage indicator into a residue specific one. This approach allows opens the way for to a detailed understanding of how crystallographic details of disulfide cleavage can be related to those occurring at physiological conditions.

At the lowest pH studied (pH 5.0), aggregation became dominant at higher absorbed doses. This was not observed at high pH conditions. The mechanism underlying the aggregation is unclear but suggests that it is charge-dependent and that X-ray damage in SAXS can be more nuanced than a completely non-specific process as previously noted in studies of lysozyme where main chain cleavage was not detected [57]. In the lysozyme case, the aggregation increased as a function of dose rate and also increased inversely to concentration. Crystallographic studies suggest that disulfide orientation and solvent exposure influence susceptibility [62]. In the lysozyme case, and for other proteins, although it is highly likely disulfide bonds are being broken, they may be buried, or other parts of the structure may constrain the overall envelope so that any structural change is not detected at the resolution of the technique [67]. Our case was designed with an easily accessible disulfide bond that when broken, gave a clear signal measurable by SAXS. For systems that have domains natively connected by exposed disulfide bonds (e.g. antibodies) [68, 69], the same process that induces a dimer monomer transition could be misinterpreted as functional dynamics in the system, underscoring a note of caution in interpretation of results and the importance of monitoring radiation damage effects in preliminary SAXS data analysis.

The diffusion present in a solution study allows transport of damaged and undamaged components which can impact the measured intensity [51]. Time also has an influence, not only in diffusion processes, but also in secondary damage due to the cascade of radicals formed. These experiments were purposely performed with a large beam size (3.4 mm^2^) to reduce the impact of diffusion. Transport and time will have an increasingly significant impact as the beam profile is reduced. Solution studies also lack crystallographic constraints, e.g. fixed lattices, close packing, and defined solvent channels, which may impede any damage processes. Our results are a snapshot of a more complex process that the crystallographic case but by the method of engineering residue specific impact that can be detected by this low-resolution technique, we open a door to the study of complex radiation chemistry phenomena in biological solution systems.

The doses used for X-ray crystallography are typically in the kilo-gray to mega-gray range. While doses used in biomedical applications such as radiotherapy and imaging are 1–2 orders of magnitude lower, structural mechanisms that occur in these settings can be studied with SAXS by utilizing low-dose collection strategies [17]. Here, the engineered protein approach allowed us to link disulfide breakage seen in crystallographic studies to damage in solution with X-ray doses closer to those that impact human health. There is considerable potential to extend our approach and look at specific damage to other residues and develop X-ray controlled protein switches. For example, visible light-based control of engineered proteins has been successfully used to modulate protein localization [70], enzyme activity [71], and gene expression [72]. These same tools, but controlled by X-rays, could augment investigations of cellular response to radiation exposure and develop treatment and mitigation strategies. While not providing the same fidelity as crystallographic approaches, and still ignoring the impact of radical formation of non-water species, this novel combination of the crystallographic and SAXS opens up a means to study and understand low dose effects, harnessing the decades of research in understanding X-ray radiation damage in a structural context.

## Supporting information

S1 Fig All buffer subtracted scattering patterns of the dimer used in the analysis. Four pH values were tested (a: pH 5, b: pH 6, c: pH 7.5, d: pH 9) at three concentrations each (from top to bottom: 5 mg/ml (replicate 1), 5 mg/ml (replicate 2), 2.5 mg/ml, and 1.25 mg/ml) across 33 exposures. The color gradient corresponds to the magnitude of the absorbed dose (36.3 Gy-1.2 kGy) delivered across 33 x 0.3 sec exposures where yellow is low dose and black is high dose.
(PNG)

S2 Fig All buffer subtracted scattering patterns of the monomer used in the analysis. Four pH values were tested (a: pH 5, b: pH 6, c: pH 7.5, d: pH 9) at three concentrations each (from top to bottom: 5 mg/ml, 5 mg/ml, 2.5 mg/ml, and 1.25 mg/ml) across 33 exposures. The color gradient corresponds to the magnitude of the absorbed dose (36.3 Gy-1.2 kGy) delivered across 33 x 0.3 sec exposures where yellow is low dose and black is high dose.
(PNG)

S3 Fig Uncropped images of the purified protein resolved on SDS-PAGE. In (a) the protein was run in the absence of a reducing agent and in (b) the protein was incubated with DTT to reduce the disulfides prior to performing PAGE. Odd numbered lanes correspond to a molecular weight ladder and even numbered lanes correspond to increasing amounts of protein sample. For the non-reducing the amount of protein was loaded in order: 1.25, 2.50, 5.0, and 10 ug, and for the reducing: 0.63 ug, 1.25 ug, 2.5 ug, and 5.0 ug. Lanes with a red X above them correspond to portions of the gel not included in Fig 1.
(PDF)

S4 Fig Calculated scattering of crystal structure models. The arrow indicates an isoscattering point that is predicted between the monomer and dimer structures at *q* ~ 0.025 Å^-1^. The small difference in rotation observed in the dimer crystal structures is distinguishable throughout the scattering curve.
(PNG)

S5 Fig Buffer intensity does not change during irradiation. The total integrated intensity was calculated for the buffer alone at each pH value tested. Error bars represent the standard deviation from integrating across the scattering curve across the upper and lower bounds of errors in *I*(*q*).
(PNG)

S6 Fig Dimensionless Kratky plots of endoH_CYS_ at different pH values. Arrows indicate the direction of dose dependent shifts in the shape of the plot. Protein was at 5 mg/ml.
(PNG)

S7 Fig pH does not affect the initial scattering of the dimer component. The dimer component alone at each pH was developed by subtracting the VF-weighted contribution of the experimental scattering of the monomer alone collected at the same dose (36.3 Gy), pH value, and concentration (5.0 mg/ml).
(PNG)

S8 Fig *R*_*g*_ and *I*(0) analysis of the monomer alone indicate no systematic changes from radiation exposure. Experimental scattering data of the monomer (reduced disulfides) was collected at four pH values (a: pH 5, b: pH 6, c: pH 7.5, d: pH 9) and three concentrations each (from top to bottom: 5 mg/ml, 2.5 mg/ml, and 1.25 mg/ml) across 33 exposures.
(PNG)

S9 Fig SVD analysis of the scattering of the monomer alone shows that the sample contains the same number of components at each pH value. Singular value decomposition analysis (SVD) was conducted on data collected with a 5.0 mg/ml protein concentration for each pH value: (a) 5.0, (b) 6.0, (c), 7.5, (d), 9.0.
(PNG)

S10 Fig The experimental scattering of the monomer is in good agreement with the crystal structure. The first exposure (36.3 Gy) of the monomer at each pH and 5.0 mg/ml was compared the calculated scattering of the monomer crystal structure (PDB 1EDT) using CRYSOL. *χ*^2^ represents the goodness of fit between the experimental and calculated scattering.
(PNG)

S11 Fig The dimer component at low doses is distinct from individual orientations in the crystal structure. In (a) the dimer component at 36.3 Gy was isolated by subtracting the volume fraction weighted scattering of the monomer crystal structure. Comparison of the scattering of this dimer component and the dimers from the crystal structure show good agreement at high-*q* but poor fitting at low-*q* suggesting that the orientation is slightly different in solution. In (a) *ab initio* modelling of SAXS data for the dimer component was performed using DENSS. The model as shown was contoured so that the volume of the envelope was close to the Porod volume as measured from the scattering data (8.0 x 10^3^ Å^3^). The final model averaged from 100 independent reconstructions with P2 symmetry (blue line) has a resolution of 30.7 Å as determined from a 0.5 FSC cutoff (red line). Front (b) and side (c) views show that the electron density can accommodate two monomeric units.
(PNG)

S12 Fig Visual comparison between *ab* initio electron density reconstruction and bead modelling from solution scattering data. The same data was used for both modelling approaches: the first exposure (36.3 Gy) collected at pH 7.5 and 5.0 mg/ml protein concentration with the volume fraction weighted monomer component subtracted. In (a) the bead model presented was the filtered model from averaging fifteen independent reconstructions assuming P2 symmetry and produced with DAMMIN [73]. In (b) an electron density model was produced with DENSS as described in S11 Fig. Two copies of the monomeric crystal structure (PDB 1EDT) were docked into the respective models using the sequential fitting tool in UCSF Chimera [34].
(PNG)

S13 Fig EOM modelling explains the experimental data better than OLIGOMER modelling at pH 7.5. In (a) the fits from modelling with OLIGOMER [43] are shown where the volume fraction weighted contributions of the monomer and dimer components are determined. The first exposure (36.3 Gy) of the monomer at pH 7.5 and 5.0 mg/ml was used as the monomer component. The dimer component was developed by subtracting the VF weighted (MW based) monomer component from the scattering of the mixture at 36.3 Gy. Fits of the VF weighted components (orange) are compared to the experimental scattering (black). Residuals are shown in (b) where the black lines are references for perfect fits between the experimental scattering and fitted models (where residuals are ~0). The fits from EOM modelling are shown in (c) and residuals are shown in (d). Ensemble models are able to satisfy the experimental scattering at all *q* values. The EOM modelling was also done using the average of two independent series of exposures at pH 7.5 at 5 mg/ml.
(PNG)

S14 Fig The rate of fragmentation is both dose and conformation dependent. Three points along (a) *D*_max_ and (b) *R*_g_ distributions from EOM were monitored for dose-dependent changes in magnitude and fit (solid black, dotted and dashed) to a logarithmic regression to determine rates of change.
(PNG)

S15 Fig The dose driven change in the conformational ensemble of the dimer was similar at pH 7.5 and 9.0. Differences in the *R*_g_ (a-c) and *D*_max_ (e-f) distributions between pH 7.5 (blue) and 9.0 (red) were monitored at three dose points: 36.3 Gy, 181.3 Gy, and 1.2 kGy. The distribution of the random pool of structures is shown in black. For both pH values, the data used for modelling was averaged from the two replicates collected with a 5.0 mg/ml protein concentration.
(PNG)

S1 Movie Movie of dose-dependent radius of gyration distribution progression from EOM modelling. (GIF)

S2 Movie Movie of dose dependent *D*_max_ distribution progression from EOM modelling. (GIF)

S1 Table Data-collection and refinement statistics. (DOCX)

S2 Table Parameters for X-ray diffraction weighted dose calculations for SAXS experiments using RADDOSE-3D. (DOCX)

S3 Table SAXS data collection parameters, analysis software employed and deposition. (DOCX)

S4 Table Experimental determined parameters from SAXS analysis endoH_CYS_ at pH 7.5 and 5 mg/ml. Values represent averages and errors are the standard deviation between two identical replicates.
(DOCX)

S5 Table Coefficients from fits of an exponential (first order) function to the VF trajectories. (DOCX)

S1 Data (GZ)
