# Supplementary figures and images for: SAXS studies of X-ray induced disulfide bond damage: Engineering high-resolution insight from a low-resolution technique
Source: PLoS One. 2020 Nov 17;15(11):e0239702. doi: 10.1371/journal.pone.0239702 (PMC7671560; doi:10.1371/journal.pone.0239702)

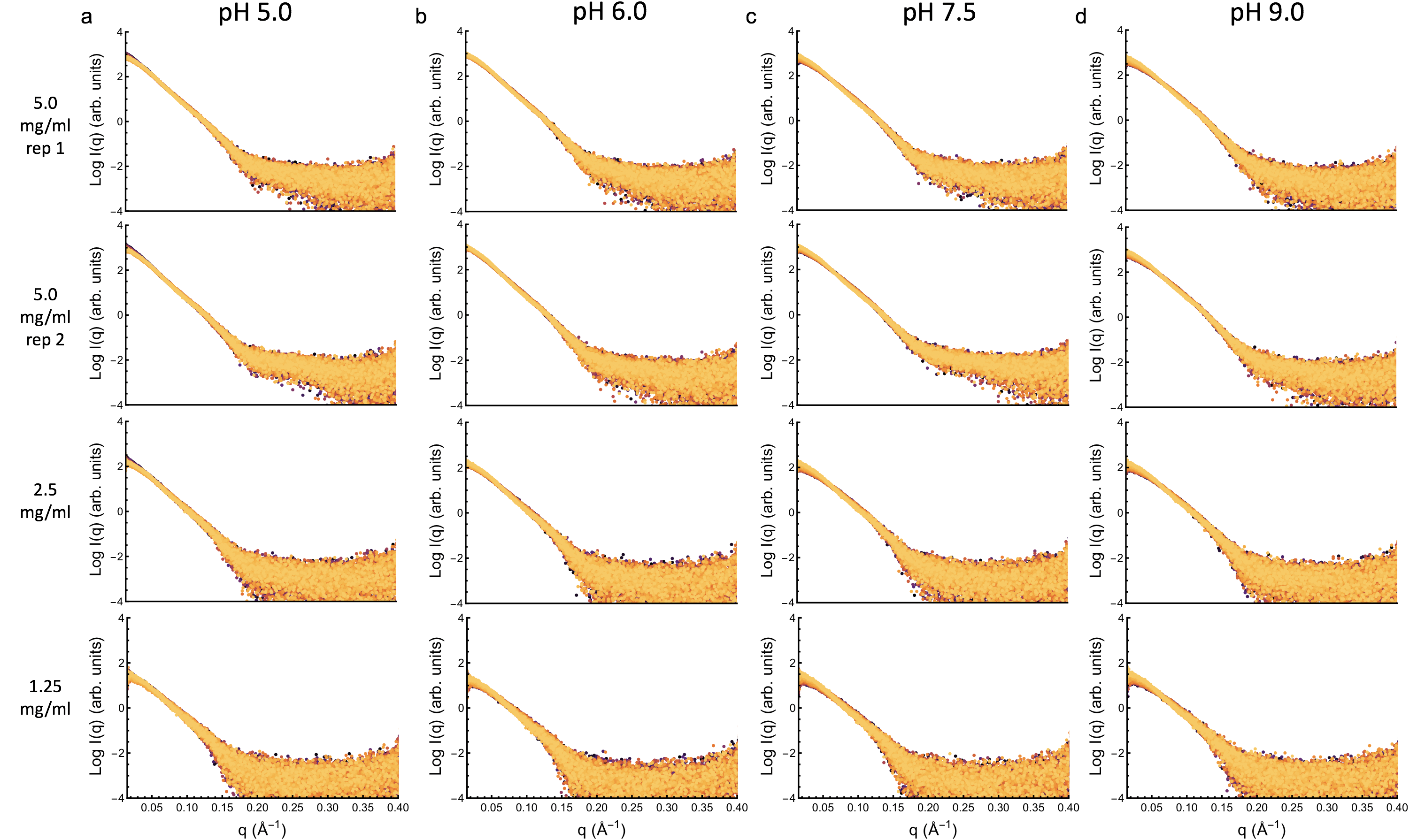

Supplement: S1 Fig — Four pH values were tested (a: pH 5, b: pH 6, c: pH 7.5, d: pH 9) at three concentrations each (from top to bottom: 5 mg/ml (replicate 1), 5 mg/ml (replicate 2), 2.5 mg/ml, and 1.25 mg/ml) across 33 exposures. The color gradient corresponds to the magnitude of the absorbed dose (36.3 Gy-1.2 kGy) delivered across 33 x 0.3 sec exposures where yellow is low dose and black is high dose. (PNG) [file pone.0239702.s001.png]

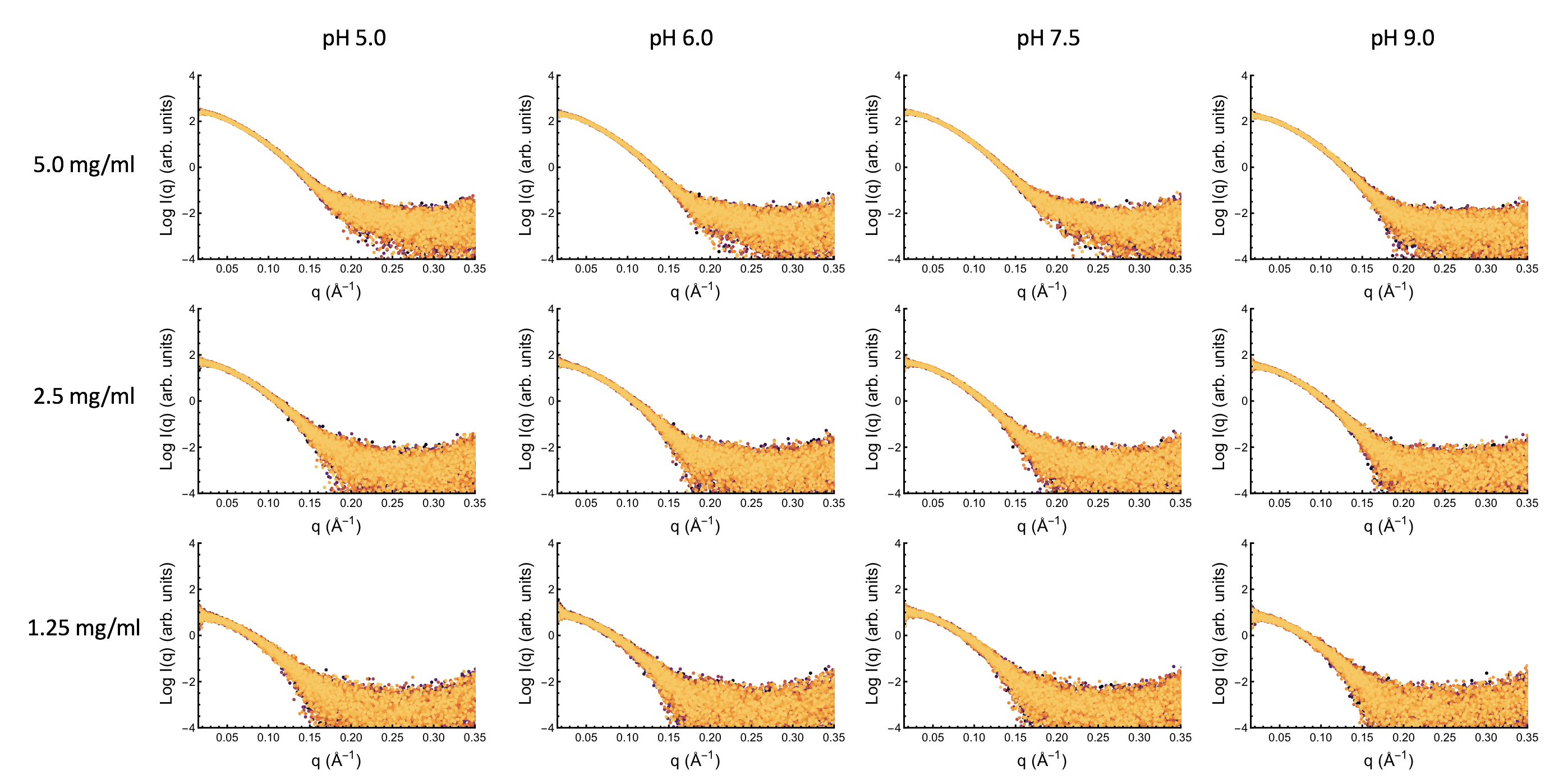

Supplement: S2 Fig — Four pH values were tested (a: pH 5, b: pH 6, c: pH 7.5, d: pH 9) at three concentrations each (from top to bottom: 5 mg/ml, 5 mg/ml, 2.5 mg/ml, and 1.25 mg/ml) across 33 exposures. The color gradient corresponds to the magnitude of the absorbed dose (36.3 Gy-1.2 kGy) delivered across 33 x 0.3 sec exposures where yellow is low dose and black is high dose. (PNG) [file pone.0239702.s002.png]

a Non-reducing

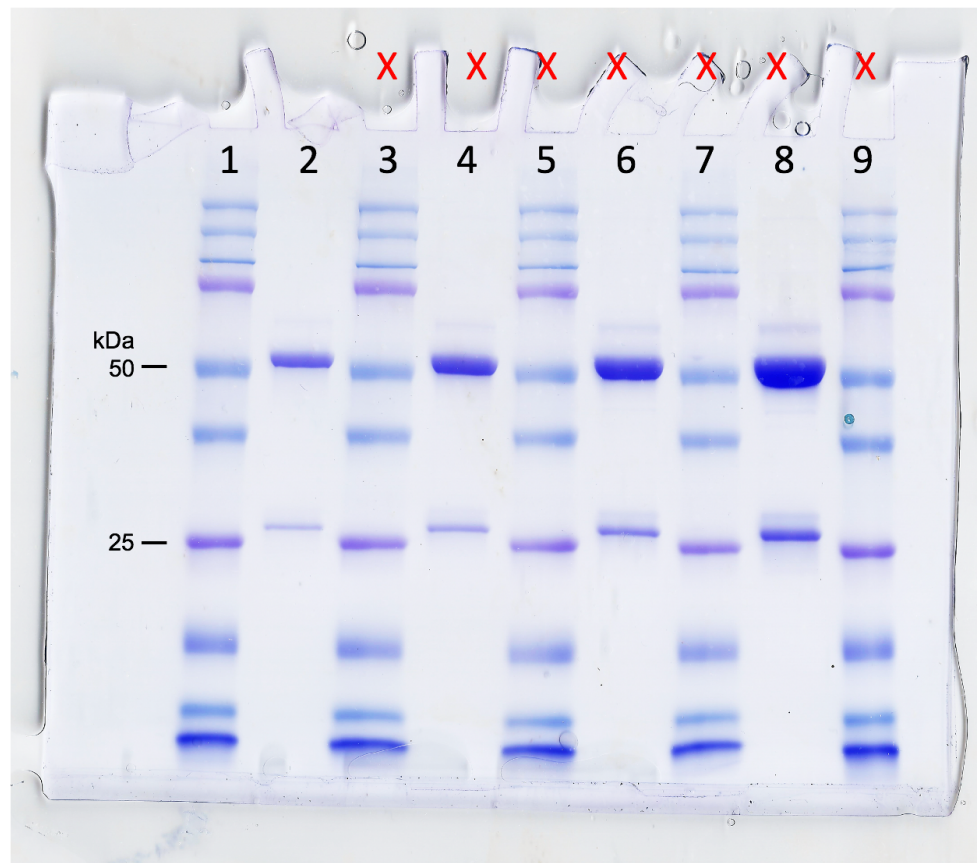

b Reducing

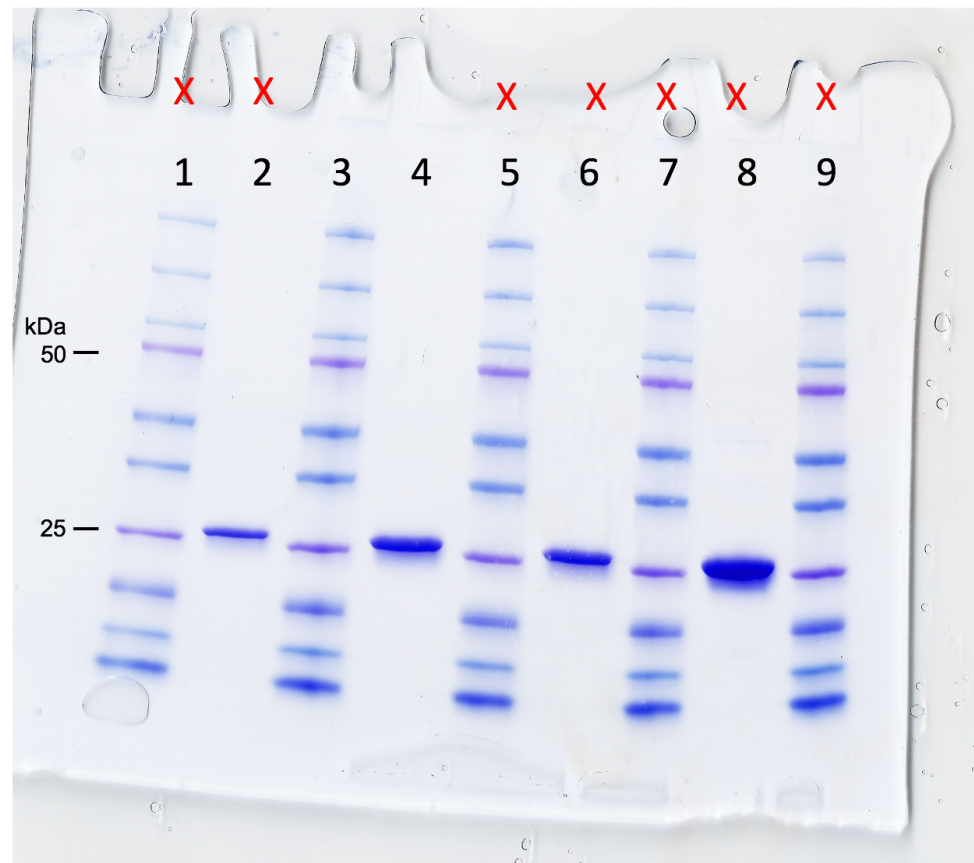

Supplement: S3 Fig — In (a) the protein was run in the absence of a reducing agent and in (b) the protein was incubated with DTT to reduce the disulfides prior to performing PAGE. Odd numbered lanes correspond to a molecular weight ladder and even numbered lanes correspond to increasing amounts of protein sample. For the non-reducing the amount of protein was loaded in order: 1.25, 2.50, 5.0, and 10 ug, and for the reducing: 0.63 ug, 1.25 ug, 2.5 ug, and 5.0 ug. Lanes with a red X above them correspond to portions of the gel not included in Fig 1. (PDF) [file pone.0239702.s003.pdf]

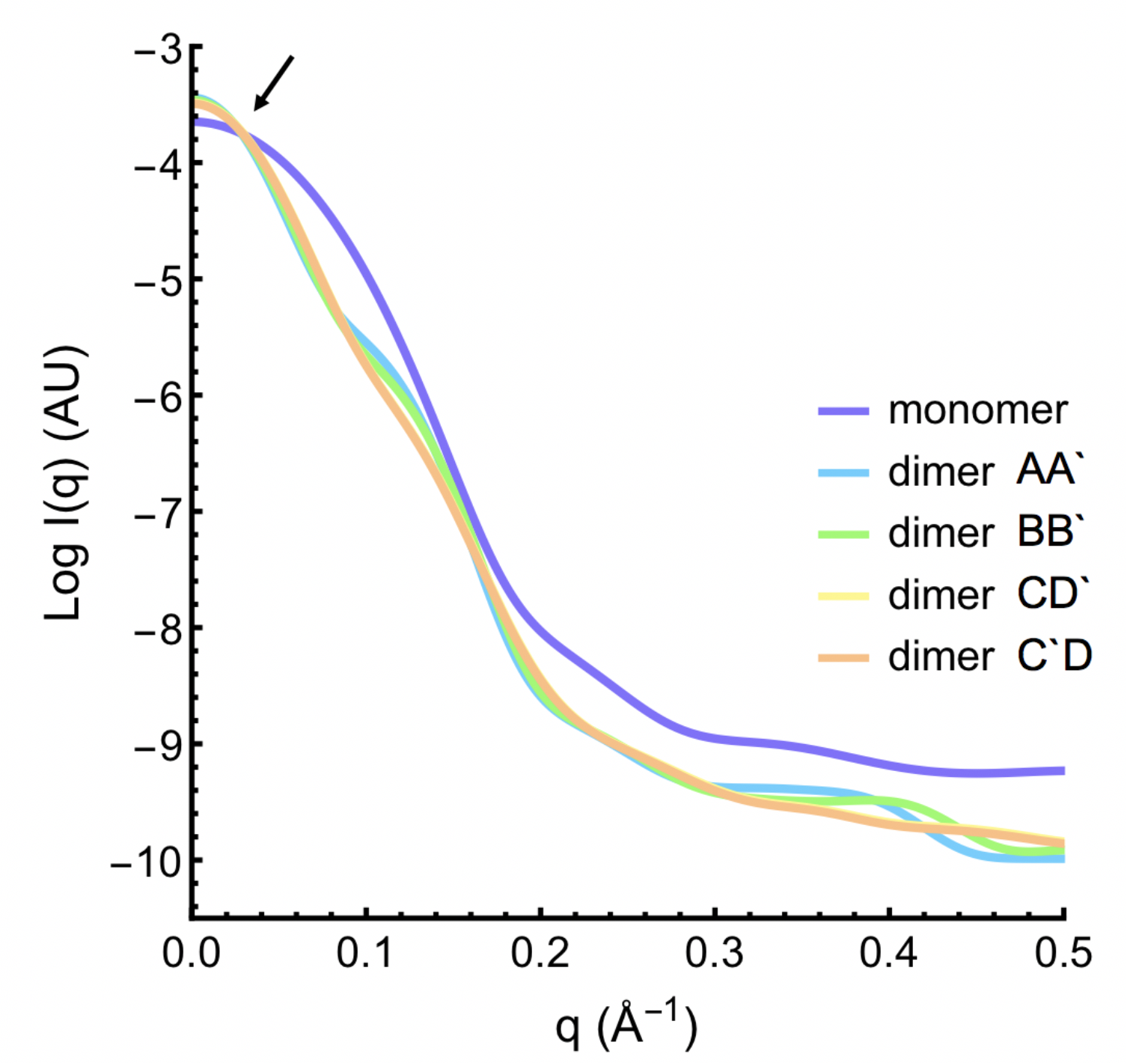

Supplement: S4 Fig — The arrow indicates an isoscattering point that is predicted between the monomer and dimer structures at q ~ 0.025 Å-1. The small difference in rotation observed in the dimer crystal structures is distinguishable throughout the scattering curve. (PNG) [file pone.0239702.s004.png]

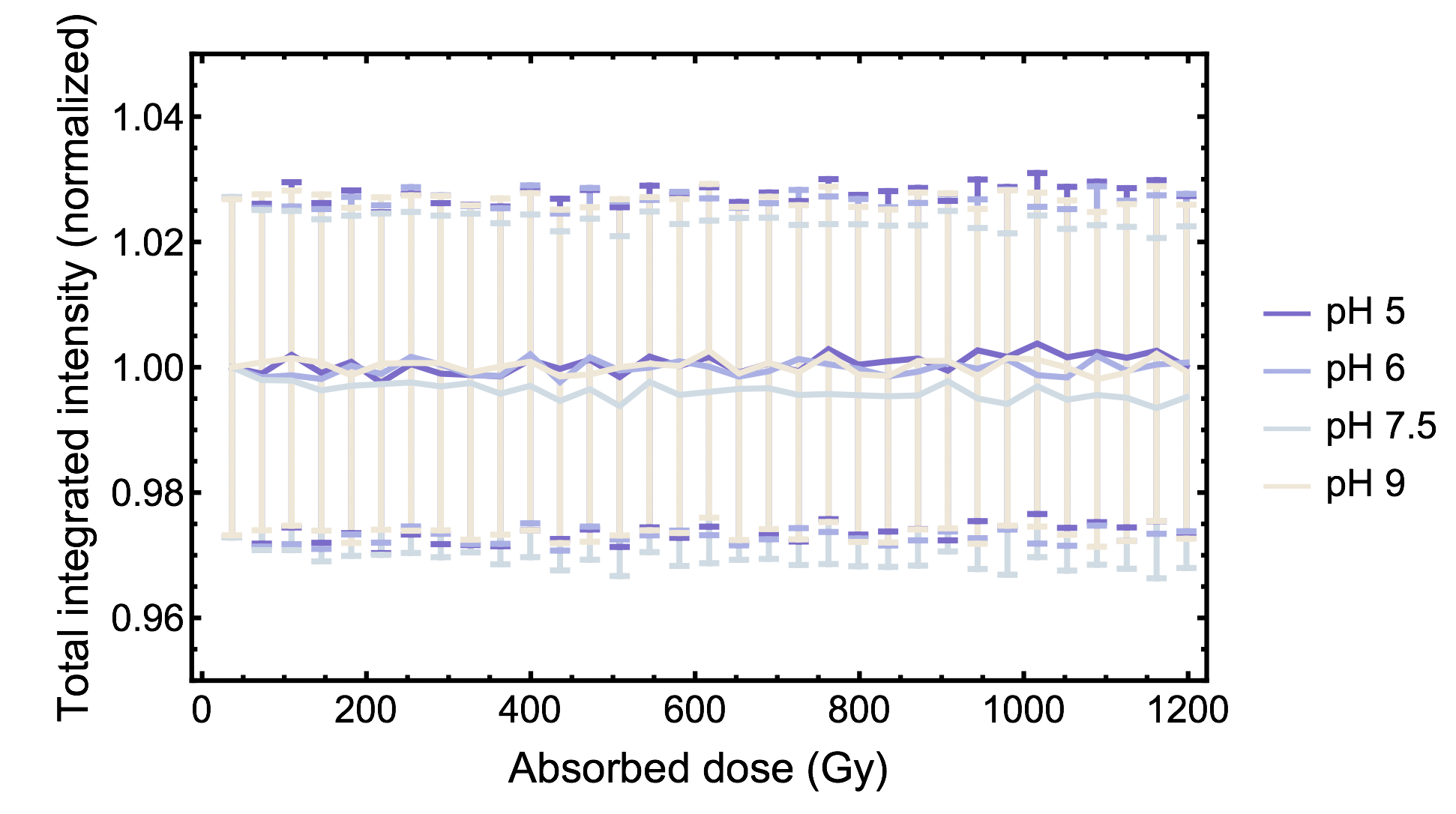

Supplement: S5 Fig — The total integrated intensity was calculated for the buffer alone at each pH value tested. Error bars represent the standard deviation from integrating across the scattering curve across the upper and lower bounds of errors in I(q). (PNG) [file pone.0239702.s005.png]

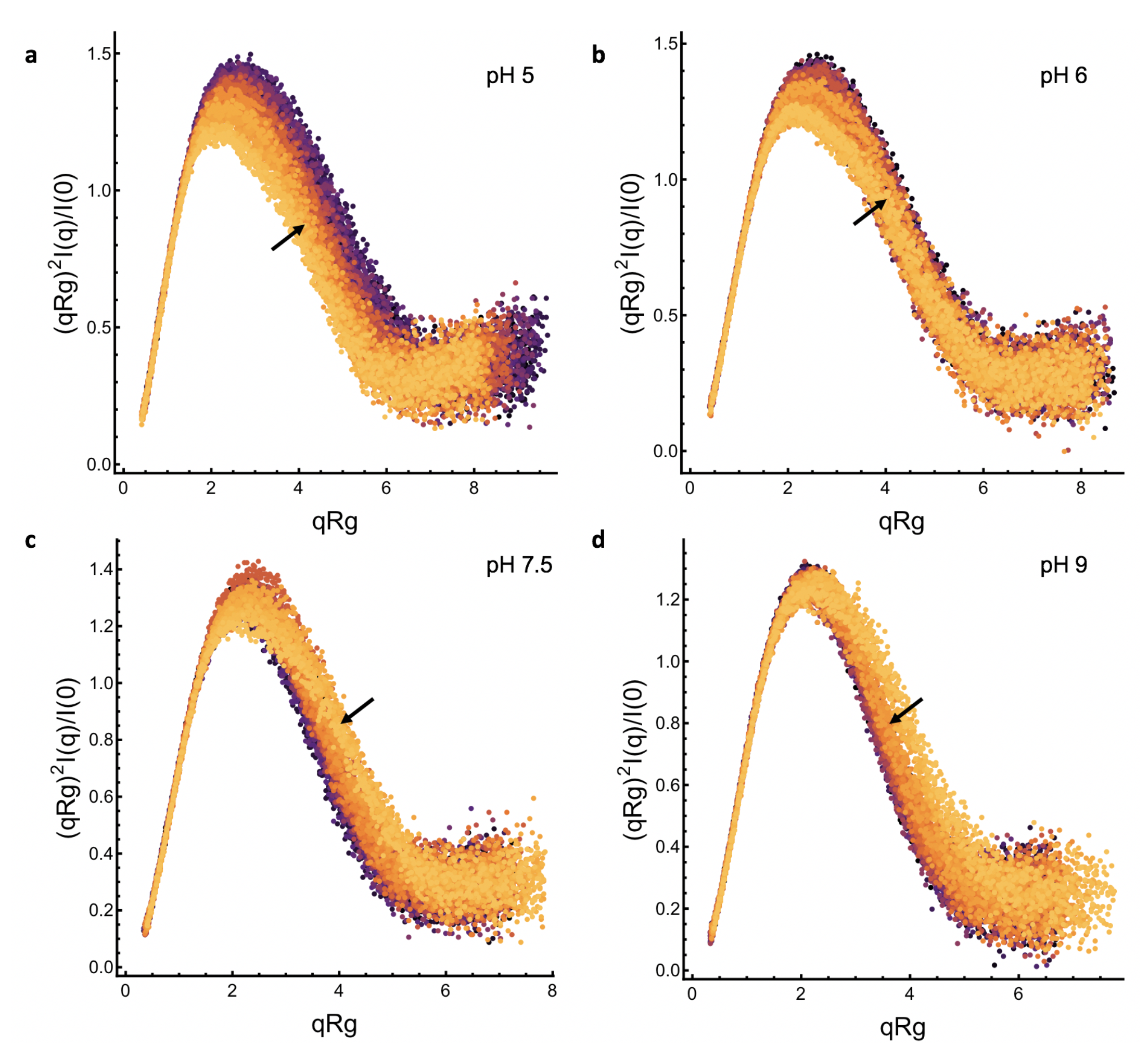

Supplement: S6 Fig — Arrows indicate the direction of dose dependent shifts in the shape of the plot. Protein was at 5 mg/ml. (PNG) [file pone.0239702.s006.png]

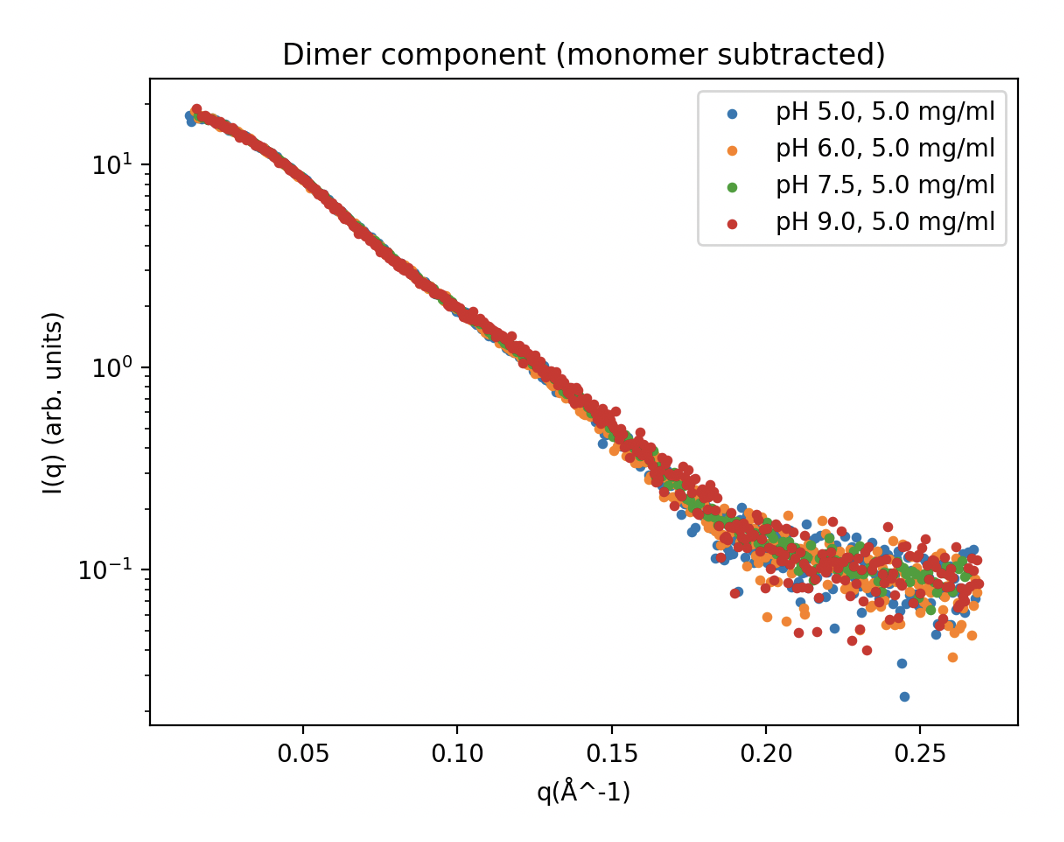

Supplement: S7 Fig — The dimer component alone at each pH was developed by subtracting the VF-weighted contribution of the experimental scattering of the monomer alone collected at the same dose (36.3 Gy), pH value, and concentration (5.0 mg/ml). (PNG) [file pone.0239702.s007.png]

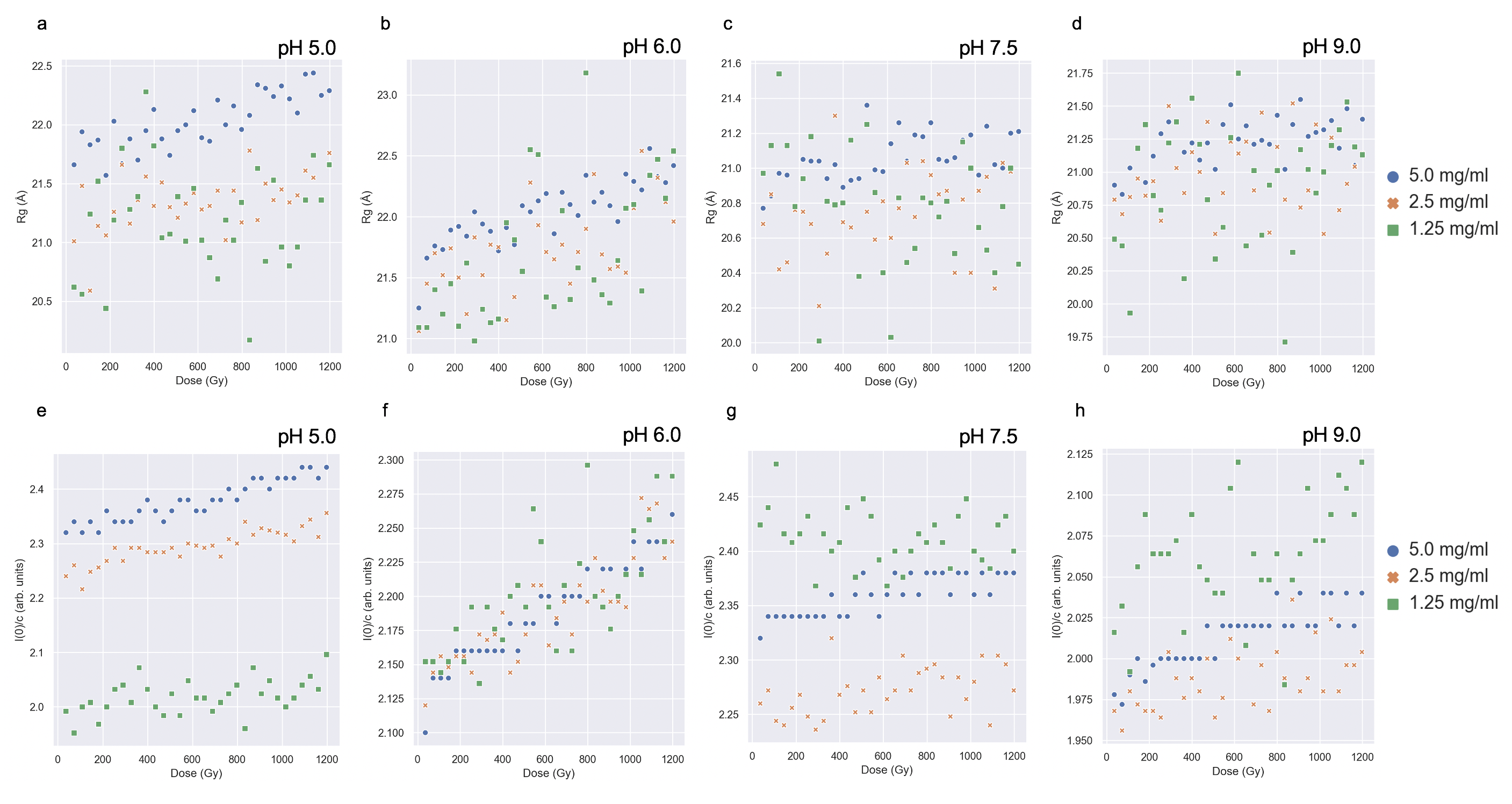

Supplement: S8 Fig — Experimental scattering data of the monomer (reduced disulfides) was collected at four pH values (a: pH 5, b: pH 6, c: pH 7.5, d: pH 9) and three concentrations each (from top to bottom: 5 mg/ml, 2.5 mg/ml, and 1.25 mg/ml) across 33 exposures. (PNG) [file pone.0239702.s008.png]

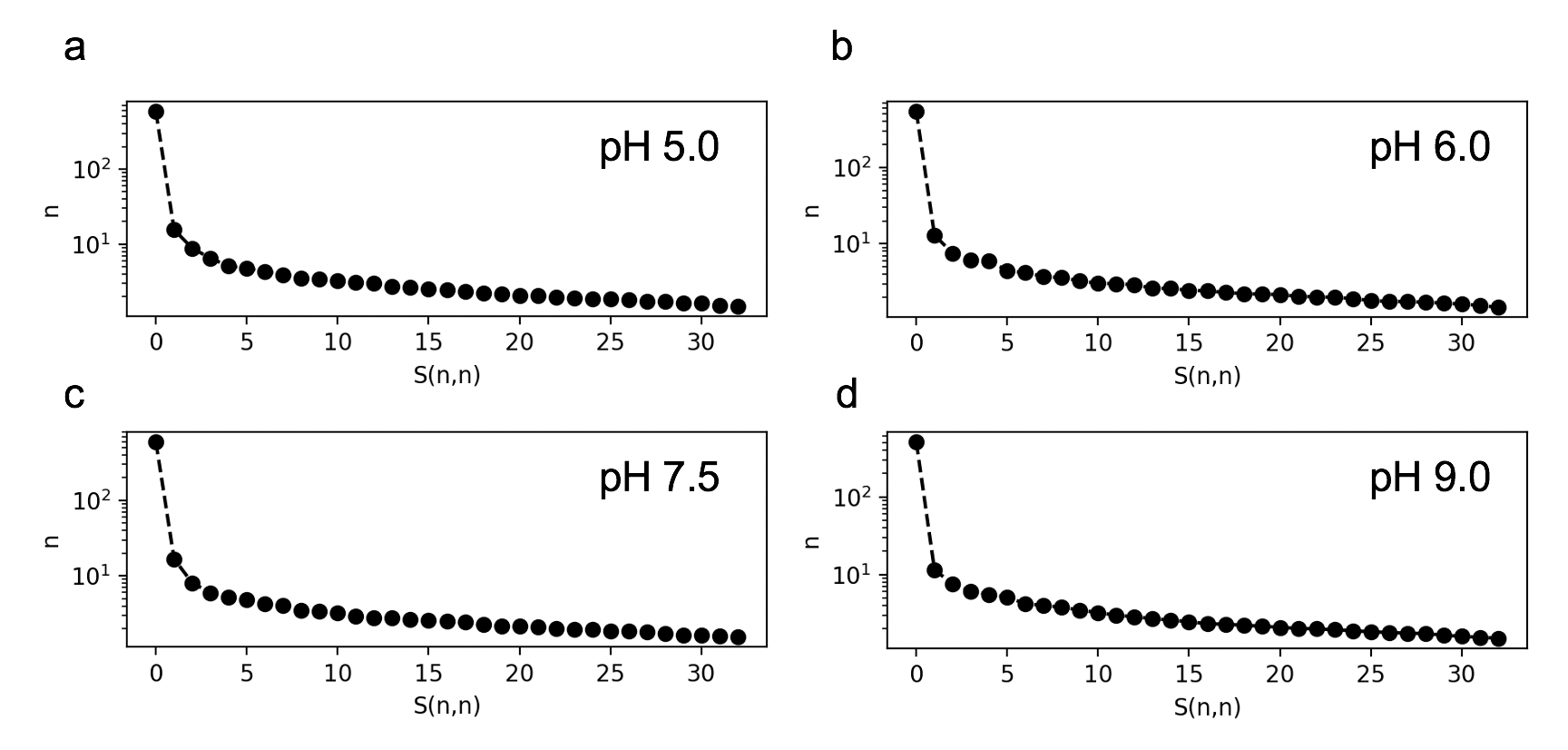

Supplement: S9 Fig — Singular value decomposition analysis (SVD) was conducted on data collected with a 5.0 mg/ml protein concentration for each pH value: (a) 5.0, (b) 6.0, (c), 7.5, (d), 9.0. (PNG) [file pone.0239702.s009.png]

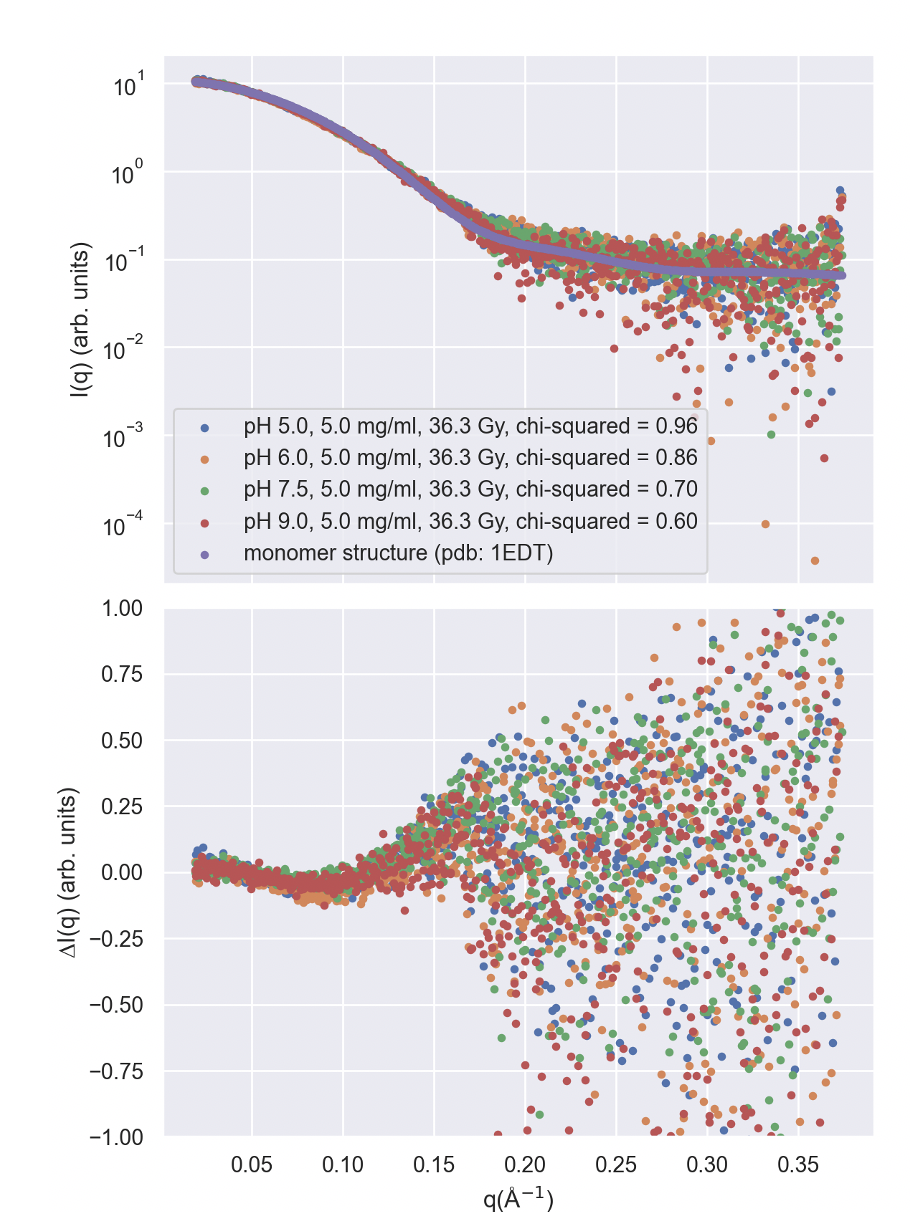

Supplement: S10 Fig — The first exposure (36.3 Gy) of the monomer at each pH and 5.0 mg/ml was compared the calculated scattering of the monomer crystal structure (PDB 1EDT) using CRYSOL. χ2 represents the goodness of fit between the experimental and calculated scattering. (PNG) [file pone.0239702.s010.png]

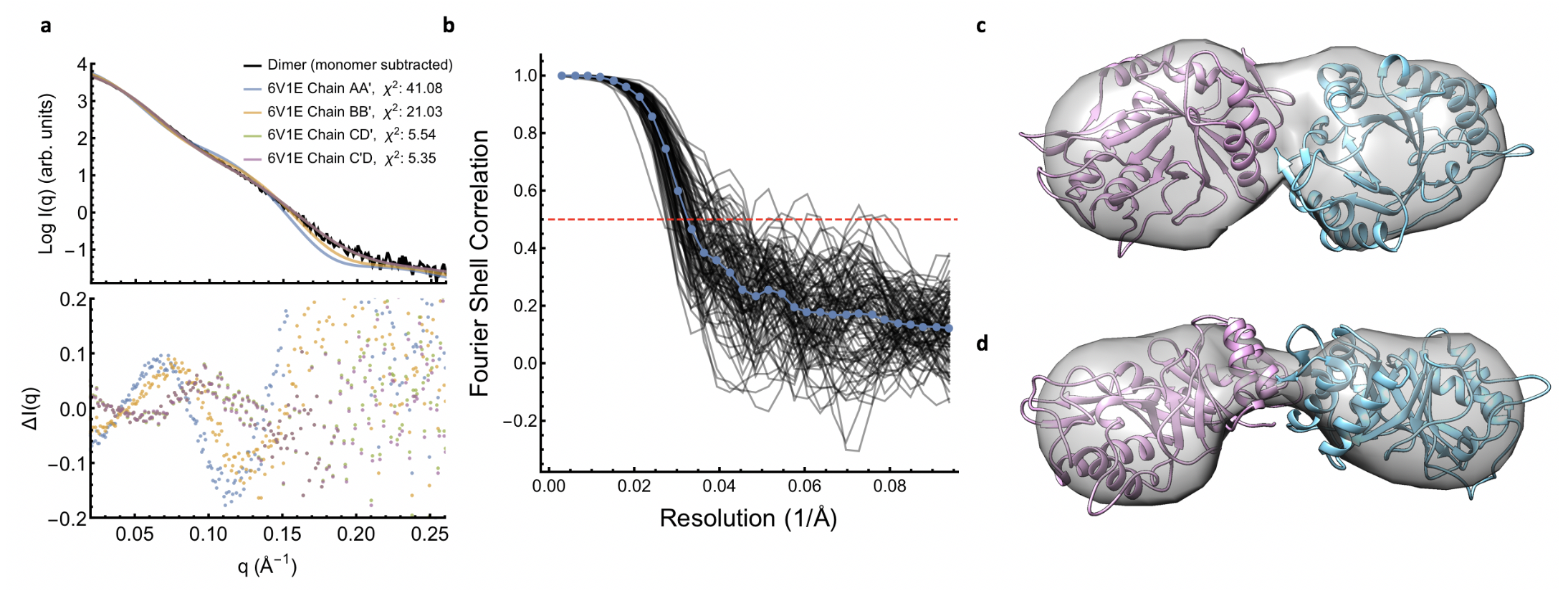

Supplement: S11 Fig — In (a) the dimer component at 36.3 Gy was isolated by subtracting the volume fraction weighted scattering of the monomer crystal structure. Comparison of the scattering of this dimer component and the dimers from the crystal structure show good agreement at high-q but poor fitting at low-q suggesting that the orientation is slightly different in solution. In (a) ab initio modelling of SAXS data for the dimer component was performed using DENSS. The model as shown was contoured so that the volume of the envelope was close to the Porod volume as measured from the scattering data (8.0 x 10^3^ Å3). The final model averaged from 100 independent reconstructions with P2 symmetry (blue line) has a resolution of 30.7 Å as determined from a 0.5 FSC cutoff (red line). Front (b) and side (c) views show that the electron density can accommodate two monomeric units. (PNG) [file pone.0239702.s011.png]

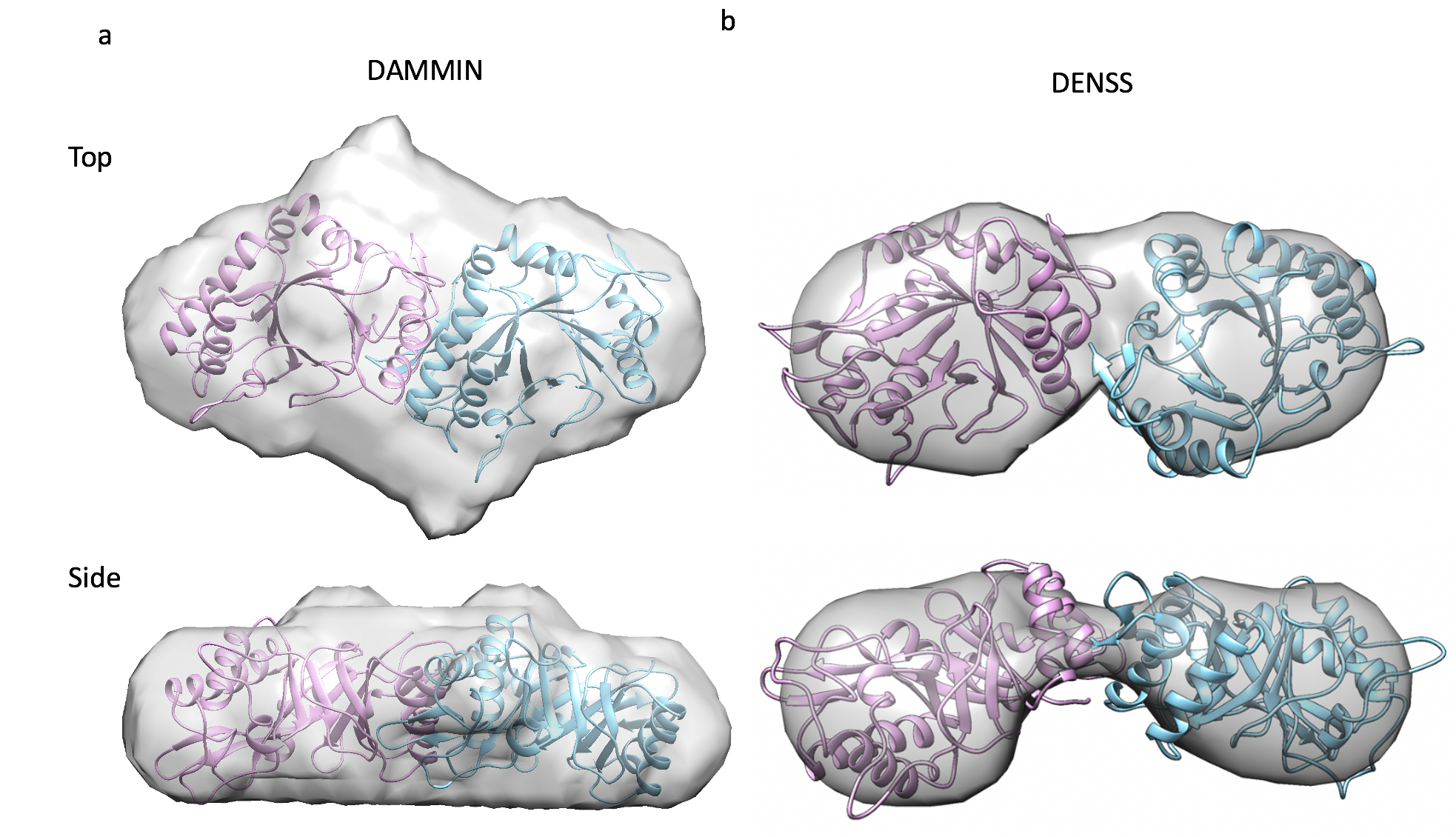

Supplement: S12 Fig — The same data was used for both modelling approaches: the first exposure (36.3 Gy) collected at pH 7.5 and 5.0 mg/ml protein concentration with the volume fraction weighted monomer component subtracted. In (a) the bead model presented was the filtered model from averaging fifteen independent reconstructions assuming P2 symmetry and produced with DAMMIN [73]. In (b) an electron density model was produced with DENSS as described in S11 Fig. Two copies of the monomeric crystal structure (PDB 1EDT) were docked into the respective models using the sequential fitting tool in UCSF Chimera [34]. (PNG) [file pone.0239702.s012.png]

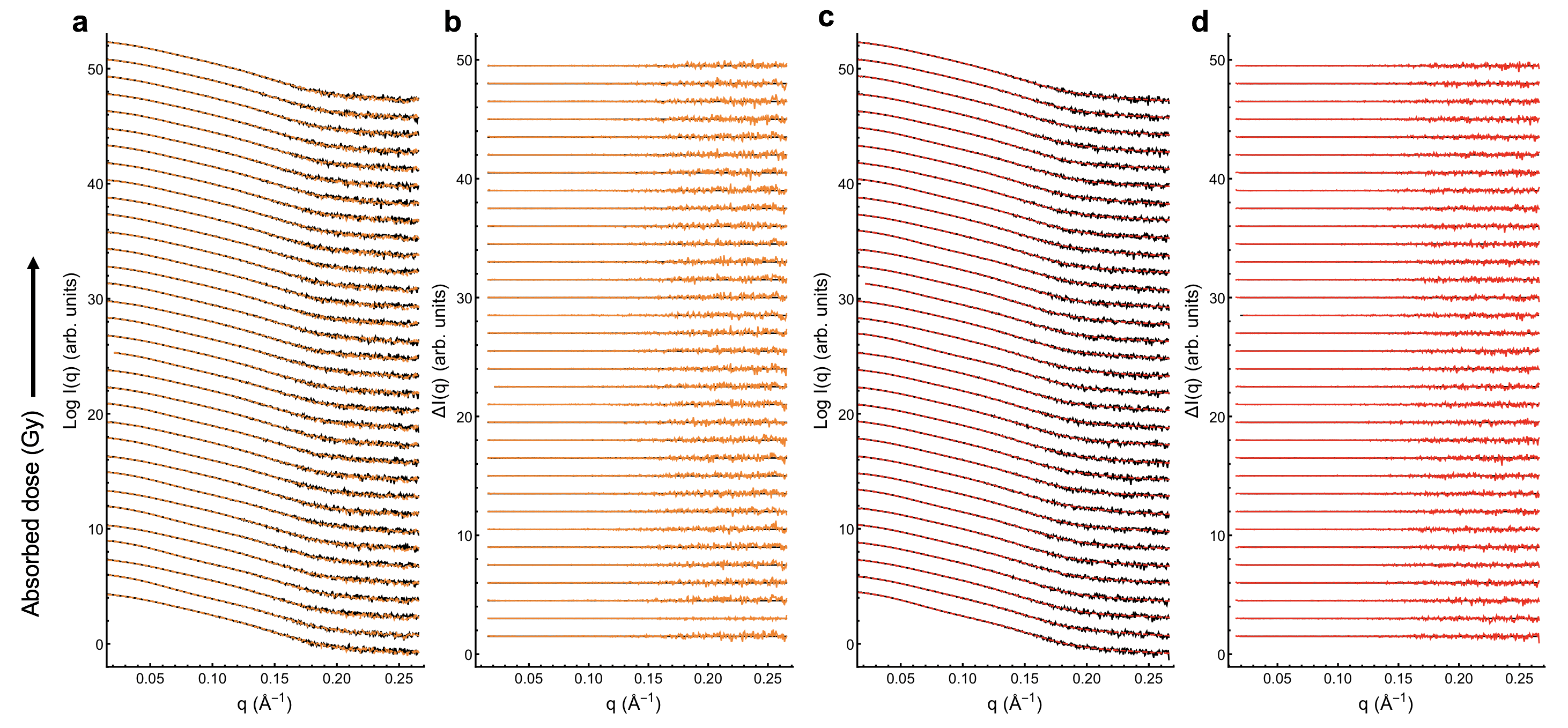

Supplement: S13 Fig — In (a) the fits from modelling with OLIGOMER [43] are shown where the volume fraction weighted contributions of the monomer and dimer components are determined. The first exposure (36.3 Gy) of the monomer at pH 7.5 and 5.0 mg/ml was used as the monomer component. The dimer component was developed by subtracting the VF weighted (MW based) monomer component from the scattering of the mixture at 36.3 Gy. Fits of the VF weighted components (orange) are compared to the experimental scattering (black). Residuals are shown in (b) where the black lines are references for perfect fits between the experimental scattering and fitted models (where residuals are ~0). The fits from EOM modelling are shown in (c) and residuals are shown in (d). Ensemble models are able to satisfy the experimental scattering at all q values. The EOM modelling was also done using the average of two independent series of exposures at pH 7.5 at 5 mg/ml. (PNG) [file pone.0239702.s013.png]

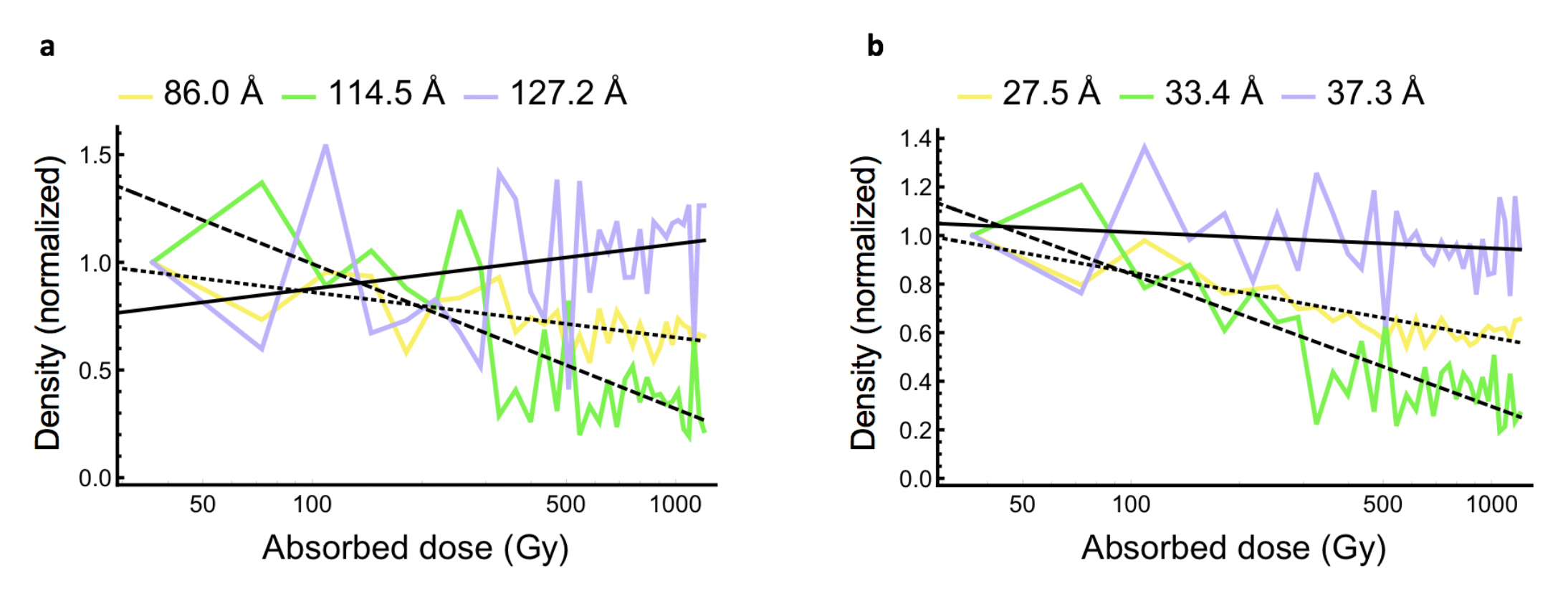

Supplement: S14 Fig — Three points along (a) Dmax and (b) Rg distributions from EOM were monitored for dose-dependent changes in magnitude and fit (solid black, dotted and dashed) to a logarithmic regression to determine rates of change. (PNG) [file pone.0239702.s014.png]

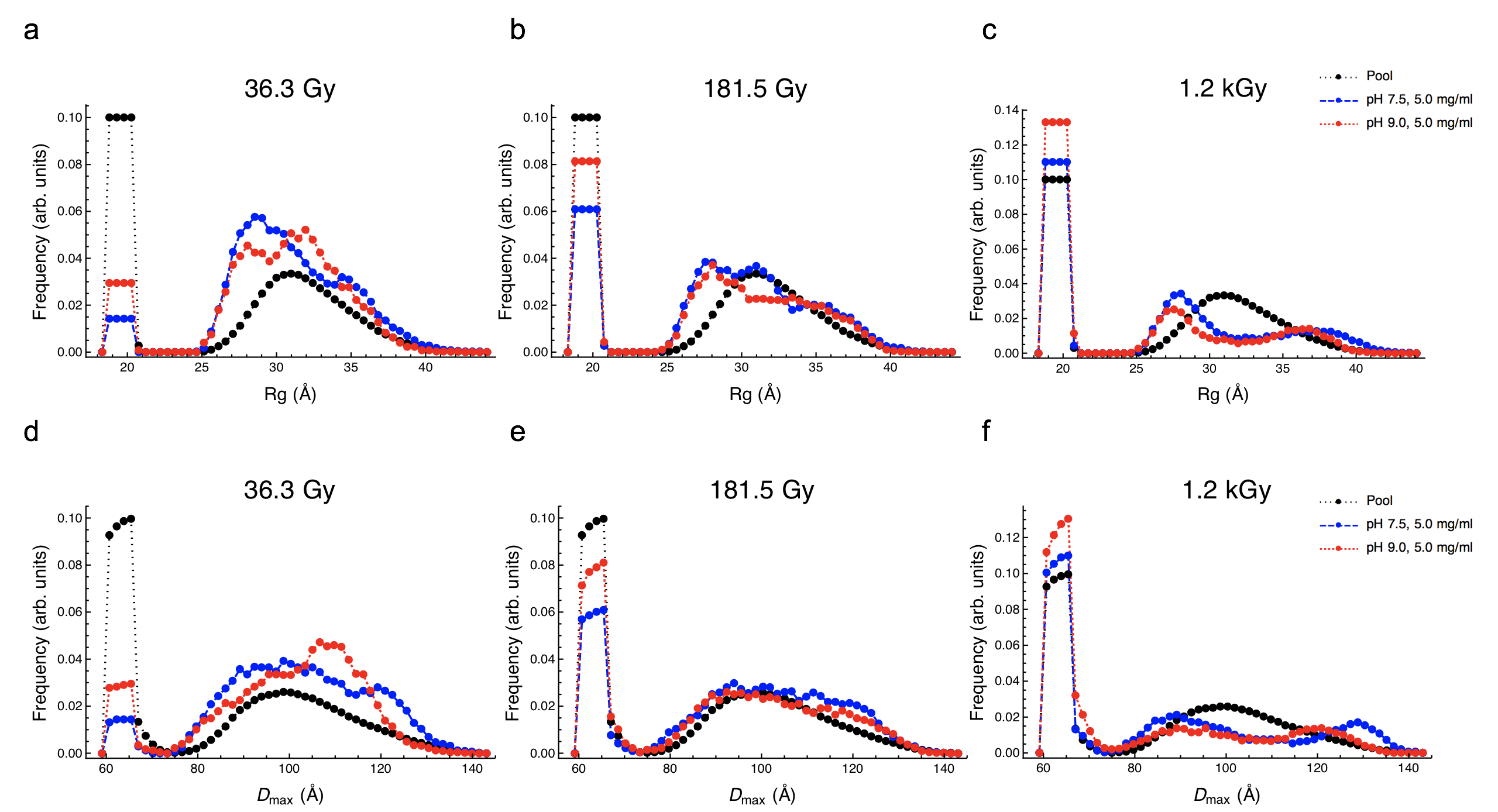

Supplement: S15 Fig — Differences in the Rg (a-c) and Dmax (e-f) distributions between pH 7.5 (blue) and 9.0 (red) were monitored at three dose points: 36.3 Gy, 181.3 Gy, and 1.2 kGy. The distribution of the random pool of structures is shown in black. For both pH values, the data used for modelling was averaged from the two replicates collected with a 5.0 mg/ml protein concentration. (PNG) [file pone.0239702.s015.png]

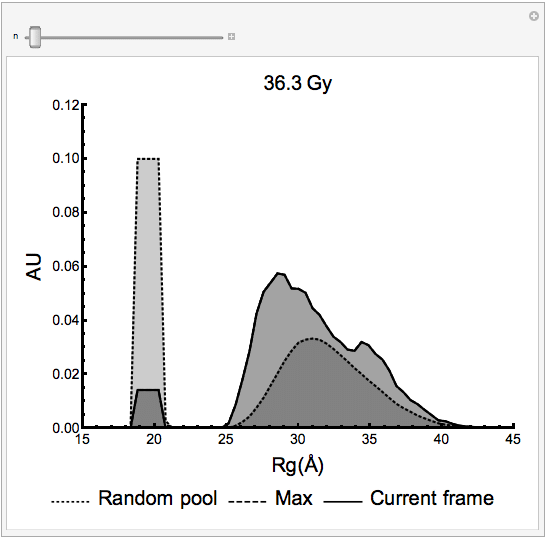

Supplement: S1 Movie — (GIF) [file pone.0239702.s016.gif]

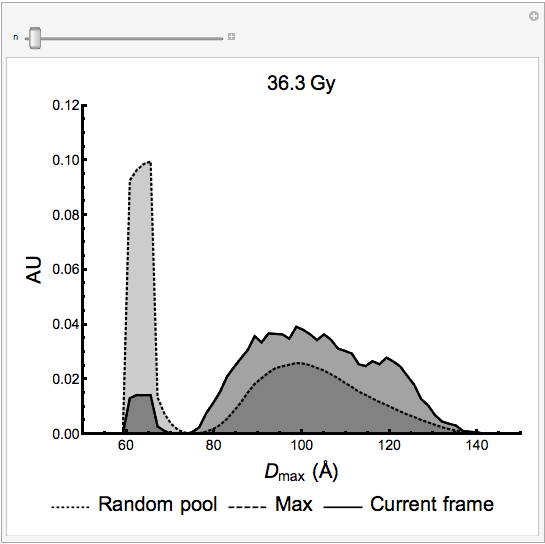

Supplement: S2 Movie — (GIF) [file pone.0239702.s017.gif]
