# Supplementary material for: SAXS studies of X-ray induced disulfide bond damage: Engineering high-resolution insight from a low-resolution technique
Source: PLoS One. 2020 Nov 17;15(11):e0239702. doi: 10.1371/journal.pone.0239702 (PMC7671560; doi:10.1371/journal.pone.0239702)
Supplement: S1 Table — (DOCX) [file pone.0239702.s018.docx]

**S1 Table. Data-collection and refinement statistics.**

Data Collection

| Diffraction source | IMCA-CAT, APS, ANL |
| --- | --- |
| Detector  Rotation range per image  Total rotation range (˚)  Temperature (K)  Wavelength (Å)  Reflections (measured/unique)  Space group  *a, b, c* (Å)  *α, β, γ* (˚)  Resolution (Å)  *R*_p.i.m._  Mean I/σ(I)  CC_1/2_  Completeness (%)  Multiplicity | PILATUS 6M  0.25  90  100  1.0  147421/74108 (14528/7330)  P2_1_22_1_  93.50, 99.50, 135.10  90, 90, 90  40.58 – 2.10 (2.18 – 2.10)  0.0948 (0.452)  5.89 (2.63)  0.986 (0.618)  99.69 (99.70)  2.0 (2.0) |

Refinement

|  |  |
| --- | --- |
| *R*_work_/*R*_free_  Reflections in working set  Reflections in test set  Total No. of atoms  Average B factor (Å^2^)  R.m.s deviations  Bond angles (˚)  Bond lengths (Å)  Ramachandran plot  Favored (%)  Allowed (%)  Outliers (%)  Molecules in asymmetric unit  PDB [1] code | 0.228 (0.327) / 0.264 (0.352)  73964 (7317)  3647 (364)  9253  14.57  0.77  0.008  96.04  3.77  0.19  4  6VE1 |

Values in Parentheses are for the outermost shell.

**References**

1. Berman, HM, Westbrook, J, Feng, Z, Gilliland, G, Bhat, TN, Weissig, H, Shindyalov, IN, Bourne, PE. The Protein Data Bank. Nucleic Acids Research, 2000, 28: 235-242.
