# Supplementary material for: SAXS studies of X-ray induced disulfide bond damage: Engineering high-resolution insight from a low-resolution technique
Source: PLoS One. 2020 Nov 17;15(11):e0239702. doi: 10.1371/journal.pone.0239702 (PMC7671560; doi:10.1371/journal.pone.0239702)
Supplement: S2 Table — (DOCX) [file pone.0239702.s019.docx]

**S2 Table. Parameters for X-ray diffraction weighted dose calculations for SAXS experiments using RADDOSE-3D.**

| Flux (ph/s) | 2.04 x 10^12^ |
| --- | --- |
| Sample container window (µm)  Sample container material  Attenuation by sample container (%)  Beam type  Beam size (mm^2^)  Energy (keV) | 20  mica  10  Top-hat  3.4  11 |
