# Supplementary material for: SAXS studies of X-ray induced disulfide bond damage: Engineering high-resolution insight from a low-resolution technique
Source: PLoS One. 2020 Nov 17;15(11):e0239702. doi: 10.1371/journal.pone.0239702 (PMC7671560; doi:10.1371/journal.pone.0239702)
Supplement: S3 Table — (DOCX) [file pone.0239702.s020.docx]

**S3 Table. SAXS data collection parameters, analysis software employed and deposition.**

| Instrument | Advanced Light Source SIBYLS SAXS Beamline with 2M detector [1] |
| --- | --- |
| Wavelength (Å)  Camera length (m)  *q* measurement (Å^-1^)  Sample configuration  Sample temperature (˚C) | 1.127  2  0.015 – 0.266  static sample cell  10 |
| Data reduction | Solvent subtraction PRIMUSqt (ATSAS 2.8.3) [2] |
| Extinction coefficient  Isoelectric point  Basis analyses  Atomic structure modelling  Volume fraction modelling  Ensemble modelling  Ab initio modelling  SASBDB [9] code | ProtParam [3]  ProtParam [3]  PRIMUSqt  CRYSOL 2.8.3 [4]  OLIGOMER 7.1 [5]  EOM 2.1 [6]  DENSS 1.4.10 [7]  DAMMIN [8]  SASDHG6 |

**References**

1. Dyer KN, Hammel M, Rambo RP, Tsutakawa SE, Rodic I, Classen S, et al. High-throughput SAXS for the characterization of biomolecules in solution: a practical approach. Methods Mol Biol. 2014;1091: 245–258. doi:10.1007/978-1-62703-691-7_18

2. Franke D, Petoukhov M V, Konarev P V, Panjkovich A, Tuukkanen A, Mertens HDT, et al. ATSAS 2.8: a comprehensive data analysis suite for small-angle scattering from macromolecular solutions. J Appl Crystallogr. 2017;50: 1212–1225. doi:10.1107/S1600576717007786

3. Gasteiger E, Hoogland C, Gattiker A, Duvaud S, Wilkins MR, Appel RD, et al. Protein Identification and Analysis Tools on the ExPASy Server BT-The Proteomics Protocols Handbook. In: Walker JM, editor. Totowa, NJ: Humana Press; 2005. pp. 571–607. doi:10.1385/1-59259-890-0:571

4. Svergun D, Barberato C, Koch MHJ. CRYSOL a Program to Evaluate X-ray Solution Scattering of Biological Macromolecules from Atomic Coordinates. J Appl Crystallogr. 1995;28: 768–773. doi:10.1107/S0021889895007047

5. Konarev P V, Volkov V V, Sokolova A V, Koch MHJ, Svergun DI. PRIMUS: a Windows PC-based system for small-angle scattering data analysis. J Appl Crystallogr. 2003;36: 1277–1282. doi:10.1107/S0021889803012779

6. Tria G, Mertens HDT, Kachala M, Svergun DI. Advanced ensemble modelling of flexible macromolecules using X-ray solution scattering. IUCrJ. 2015;2: 207–217. doi:10.1107/S205225251500202X

7. Grant TD. Ab initio electron density determination directly from solution scattering data. Nat Methods. 2018;15: 191–193. doi:10.1038/nmeth.4581

8. Svergun, D. I. (1999). Biophys. J. 76, 2879–2886.

9. SASBDB reference Valentini E, Kikhney AG, Previtali G, Jeffries CM, Svergun DI. SASBDB, a repository for biological small-angle scattering data. Nucleic Acids Res. 2015;43: D357–D363. doi:10.1093/nar/gku1047
