# Supplementary material for: SAXS studies of X-ray induced disulfide bond damage: Engineering high-resolution insight from a low-resolution technique
Source: PLoS One. 2020 Nov 17;15(11):e0239702. doi: 10.1371/journal.pone.0239702 (PMC7671560; doi:10.1371/journal.pone.0239702)
Supplement: S4 Table — Values represent averages and errors are the standard deviation between two identical replicates. (DOCX) [file pone.0239702.s021.docx]

**S4 Table. Experimental determined parameters from SAXS analysis endoH_CYS_ at pH 7.5 and 5 mg/ml.** Values represent averages and errors are the standard deviation between two identical replicates.

| Dose (Gy) | Molecular weight (kDa) | Radius of gyration (Å) | I(0) (arb. units) |  |
| --- | --- | --- | --- | --- |
| 36.3 | 48.3 +/- 1.65 | 30.6 +/- 0.31 | 21.2 +/- 2.05 |  |
| 72.6 | 46.8 +/- 1.16 | 30.4 +/- 0.46 | 20.8 +/- 1.69 |  |
| 109 | 45.5 +/- 0.78 | 30.5 +/- 0.22 | 20.6 +/- 1.76 |  |
| 145 | 44.0 +/- 0.97 | 29.6 +/- 1.42 | 19.7 +/- 1.20 |  |
| 182 | 43.0 +/- 0.45 | 29.8 +/- 1.18 | 19.5 +/- 1.55 |  |
| 218 | 42.2 +/- 0.77 | 29.1 +/- 0.41 | 19.0 +/- 1.62 |  |
| 254 | 41.3 +/- 0.46 | 28.6 +/- 0.05 | 18.6 +/- 1.90 |  |
| 290 | 40.6 +/- 0.00 | 28.6 +/- 0.73 | 18.4 +/- 1.48 |  |
| 327 | 39.9 +/- 0.21 | 28.6 +/- 0.77 | 18.3 +/- 2.33 |  |
| 363 | 39.2 +/- 0.52 | 28.4 +/- 0.27 | 18.0 +/- 1.97 |  |
| 399 | 38.8 +/- 0.16 | 28.3 +/- 0.18 | 17.8 +/- 1.83 |  |
| 436 | 38.6 +/- 0.23 | 27.6 +/- 0.02 | 17.5 +/- 1.90 |  |
| 472 | 38.3 +/- 0.27 | 28.2 +/- 0.09 | 17.7 +/- 1.83 |  |
| 508 | 38.0 +/- 0.09 | 27.9 +/- 0.29 | 17.4 +/- 1.69 |  |
| 545 | 34.0 +/- 0.24 | 28.7 +/- 1.13 | 17.7 +/- 1.48 |  |
| 581 | 37.7 +/- 0.00 | 27.9 +/- 0.28 | 17.3 +/- 1.90 |  |
| 617 | 37.4 +/- 0.00 | 28.3 +/- 0.78 | 17.5 +/- 1.55 |  |
| 653 | 37.2 +/- 0.20 | 27.8 +/- 0.59 | 17.3 +/- 2.05 | |
| 688 | 34.8 +/- 3.20 | 28.1 +/- 0.21 | 17.4 +/- 1.90 | |
| 726 | 37.3 +/- 0.00 | 27.8+/- 0.02 | 17.2 +/- 1.83 | |
| 762 | 34.9 +/- 3.61 | 27.6 +/- 0.51 | 17.2 +/- 2.05 | |
| 799 | 37.3 +/- 0.01 | 27.9 +/- 0.32 | 17.2 +/- 1.62 | |
| 835 | 37.3 +/- 0.50 | 27.5 +/- 0.035 | 17.0 +/- 1.69 | |
| 871 | 37.0+/- 0.20 | 27.6 +/- 0.81 | 17.1 +/- 2.05 | |
| 908 | 37.2 +/- 0.29 | 27.3 +/- 0.95 | 16.9 +/- 2.12 | |
| 944 | 36.9+/- 0.23 | 27.3 +/- 0.07 | 16.9 +/- 1.69 | |
| 980 | 36.8 +/- 0.37 | 28.1 +/- 0.02 | 17.2 +/- 1.69 | |
| 1016 | 37.2 +/- 0.72 | 27.9 +/- 0.48 | 17.2 +/- 1.90 | |
| 1053 | 36.7 +/- 0.43 | 27.0 +/- 0.61 | 16.8 +/- 2.05 | |
| 1089 | 34.7 +/- 3.30 | 27.7 +/- 0.02 | 17.1 +/- 1.76 | |
| 1125 | 37.0 +/- 0.71 | 27.3 +/- 0.11 | 16.9 +/- 1.62 | |
| 1161 | 37.2 +/- 0.43 | 28.1 +/- 0.53 | 17.3 +/- 1.48 | |
| 1200 | 37.2 +/- 0.50 | 27.2 +/- 0.48 | 16.9 +/- 1.90 | |
