# Supplementary material for: SAXS studies of X-ray induced disulfide bond damage: Engineering high-resolution insight from a low-resolution technique
Source: PLoS One. 2020 Nov 17;15(11):e0239702. doi: 10.1371/journal.pone.0239702 (PMC7671560; doi:10.1371/journal.pone.0239702)
Supplement: S5 Table — (DOCX) [file pone.0239702.s022.docx]

**S5 Table. Coefficients from fits of an exponential (first order) function to the VF trajectories.**

| pH | Concentration (mg/ml) | ${vf}_{0}$ – initial VF_dimer_ | $k_{1}$ – rate constant (Gy^-1^) |
| --- | --- | --- | --- |
| 7.5 | 5.0 (replicate 1) | 0.766 +/- 0.012 | 0.0044 +/- 0.0002 |
| 9.0 | 5.0 (replicate 2)  2.5  1.25  5.0 (replicate 1)  5.0 (replicate 2)  2.5  1.25 | 0.879 +/- 0.014  0.754 +/- 0.024  0.684 +/- 0.064  0.781 +/- 0.015  0.794 +/- 0.020  0.683 +/- 0.028  0.534 +/- 0.081 | 0.0050 +/- 0.0002  0.0050 +/- 0.0003  0.0049 +/- 0.0007  0.0041 +/- 0.0002  0.0040 +/- 0.0002  0.0047 +/- 0.0003  0.0033 +/- 0.0007 |
